# Supplementary material for: Intrinsic hydroquinone-functionalized aggregation-induced emission core shows redox and pH sensitivity
Source: Commun Chem. 2021 Apr 23;4:55. doi: 10.1038/s42004-021-00492-4 (PMC9814920; doi:10.1038/s42004-021-00492-4)
Supplement: Supplementary file 1 — Supplementary Information [file 42004_2021_492_MOESM1_ESM.pdf]

Supplementary Information

Intrinsic hydroquinone-functionalized aggregation-induced emission core shows redox and pH sensitivity

Mengshi Wang[1+], Yuanheng Wang[2+],Renjian Hu[1], Jinying Yuan[3], Mei Tian[4], Xiaoyong Zhang[5], Zhigang Shuai[2] and Yen Wei[1,6*]

Table of Contents

1. Experimental Procedures **4**

Supplementary Note S1

Scheme S1. synthesis of **DMBTPE** and **HQTPE**.

Scheme S2. synthesis of **QTPE**.

2. Characterization **6**

Figure S1. ^{1}H NMR spectrum of **DMBTPE** in DMSO-d_6.

Figure S2. ^{13}C NMR spectrum of **DMBTPE** in DMSO-d_6.

Figure S3. Mass spectrum of **DMBTPE**.

Figure S4. ^{1}H NMR spectrum of **HQTPE** in DMSO-d_6.

Figure S5. ^{13}C NMR spectrum of **HQTPE** in DMSO-d_6.

Figure S6. Mass spectrum of **HQTPE**.

Figure S7. ^{1}H NMR spectrum of **QTPE** in DMSO-d_6.

Figure S8. Mass spectrum of **QTPE**.

Figure S9. FT-IR spectra of **HQTPE** and **QTPE**.

Figure S10. UV-vis absorption spectra of **HQTPE** and **QTPE**.

Figure S11. Fluorescence spectra of 100 μM **HQTPE** in different solvants.

Figure S12. Scanning electron micrographs of **HQTPE** particles.

Figure S13. Photographs of **HQTPE/QTPE** solid and solutions.

3. Application **14**

Figure S14. Fitting curve of the FL intensity of **HQTPE** solutions at different concentrations without **QTPE**.

Figure S15. Fitting curve of the FL intensity of **DMBTPE** solutions at different pH conditions.

Figure S16. Influence of **HQTPE** on the capture of singlet oxygen ($^1O_2$) generated from Chlorin e6.

Figure S17. Efficient response of **HQTPE** to different oxidants.

Figure S18. Photographs of **HQTPE** and **TBHQ** powder exposed to air within seven days.

Figure S19. ^{1}H NMR spectra in DMSO-d_6 of **TBHQ** solution exposed to air within seven days.

4. Computational Studies **18**

Supplementary Note 2

Figure S20. Two possible forms of mono-deprotonated anion of **HQTPE**.

Figure S21. The calculated FMO energy levels of **HQTPE** and **QTPE** (solution phase).

Table S1. The contribution percent of transitions between noted FMOs to S_0-S_1 transition in **HQTPE** and **QTPE.**

Figure S22. Proposed excited state electron transfer pathway of excited-state **HQTPE**.

Figure S23. The calculated FMO energy levels of **HQTPE** and its anion (solution phase).

Table S2. The contribution percent of transitions between noted FMOs to S_0-S_1 transition in **HQTPE** and **HQTPE Anion-1.**

Table S3. Some calculated photochemical properties of the relative compounds.

5. Supplementary References..23

Supplementary Data 1-8 contain the Cartesian Coordinates for each related compound or intermediate.

Experimental Procedures

Supplementary Note S1

1-(2,5-Dimethoxyphenyl)-1,2,2-triphenylethene (DMBTPE)[1] A Schlenk tube was charged with triphenylvinyl bromide (3.352 g, 10.0 mmol), 3-(boronic acid pinacol ester)-1,4-dimethoxybezene (3.302 g, 12.5 mmol) and potassium carbonate (3.455 g, 25.0 mmol). Toluene (40 mL) and deionised water (12.5 mL) were then added. The mixture was thoroughly degassed before adding Tetrakis(triphenylphosphine)palladium catalyst (35 mg, 0.03 mmol). The resulting mixture was stirred for 24 hours at 90 ^{o}C under an inert nitrogen atmosphere, then cooled to room temperature and extracted with ethyl acetate and washed by brine. Then the combined organic layer was filtered, concentrated and purified using column chromatography (silica gel, petroleum ether followed by PE/DCM 80:20, v/v). The pure product was a white solid with strong fluorescence (3.501g, 8.92 mmol, 89.2%).

1-Hydroquinol-1,2,2-triphenylethene (HQTPE)[2] To a solution of **DMBTPE** (1.40 g, 3.56 mmol) in anhydrous dichloromethane (30 mL) boron tribromide was slowly added (3.59 g, 14.3 mmol) drop-wise at -78 ^{o}C and then warmed to room temperature and stirred for 18 hours. The reaction was quenched by the addition of deionised water (20 mL). The organic layer was filtered and the solvent was removed via a rotary evaporator. The product was an offwhite and fluorescent solid with satisfactory yield (1.08 g, 2.97 mmol, 83.5%). Further purification could be implemented by column chromatography (silica gel, petroleum ether followed by PE/DCM 50:50, v/v).

1-Benzoquinol-1,2,2-triphenylethene (QTPE)[3] A 150 ml flask was charged with a vigorously stirred suspension of chromatographic grade silica gel (10 g) in dichloromethane (80 mL). Then a 0.65 M aq. solution of $NaIO_4$ (10 mL) was added with stirring, whence a flaky suspension was formed. **HQTPE** (1.82g, 5.0 mmol) in dichloromethane (10 mL) was then added. The reaction became colored rapidly and thoroughly completed within 5 min. The mixture was then filtered, and the silica gel was thoroughly washed with dichloromethane. The solvent was removed via a rotary evaporator. The product was a darkly red solid with satisfactory yield (1.79 g, 4.95 mmol, 98.9%).

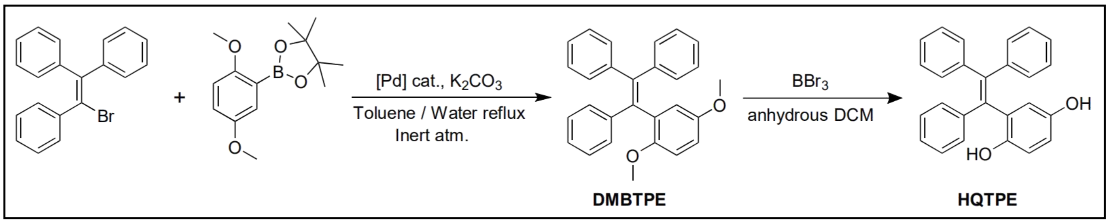

Scheme S1. synthesis of 1-(2,5-dimethoxyphenyl)-1,2,2-triphenylethene (**DMBTPE**) and 1-hydroquinol-1,2,2-triphenylethene (**HQTPE**)

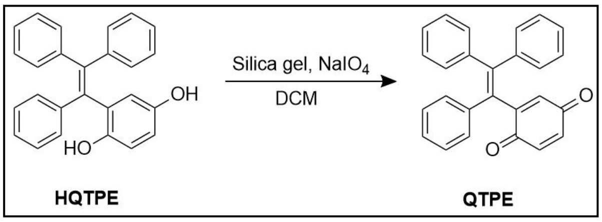

Scheme S2 synthesis of 1-benzoquinol-1,2,2-triphenylethene (**QTPE**)

Characterization

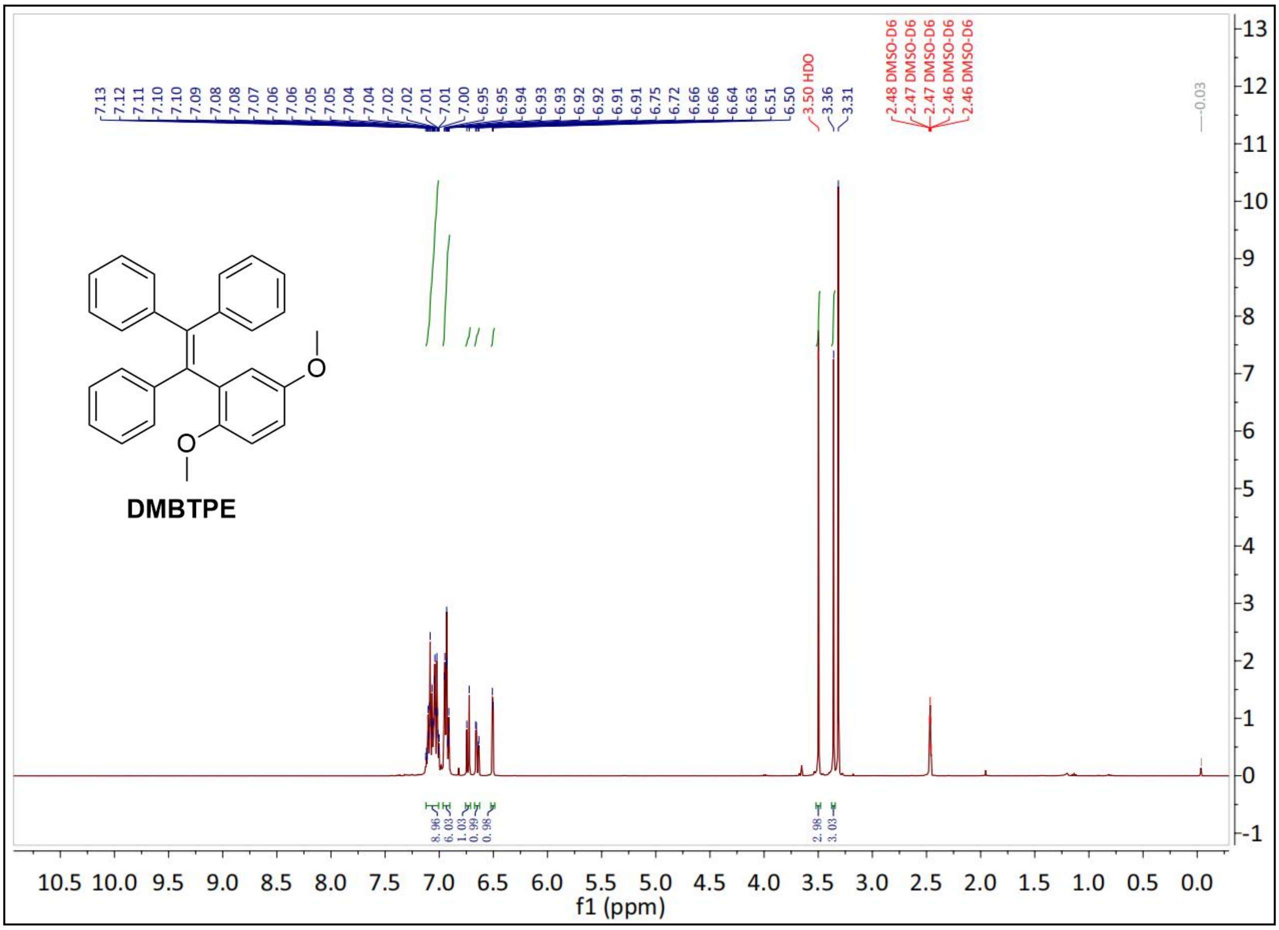

Figure S1. ^{1}H NMR spectrum of **DMBTPE** in DMSO-d_6. ^{1}H NMR (400 MHz, DMSO-d6): δ7.12-7.01 (m, 9H, of three benzene rings), 6.93 (ddd, J = 9.4, 7.6, 2.0 Hz, 6H, of three benzene rings), 6.73 (d, J = 8.9 Hz, 1H, of the DMB ring), 6.65 (dd, J = 8.8, 3.1 Hz, 1H, of the DMB ring), 6.51 (d, J = 3.1 Hz, 1H, of the DMB ring), 3.50 (s, 3H, of methoxyl), 3.36 (s, 3H, of methoxyl).

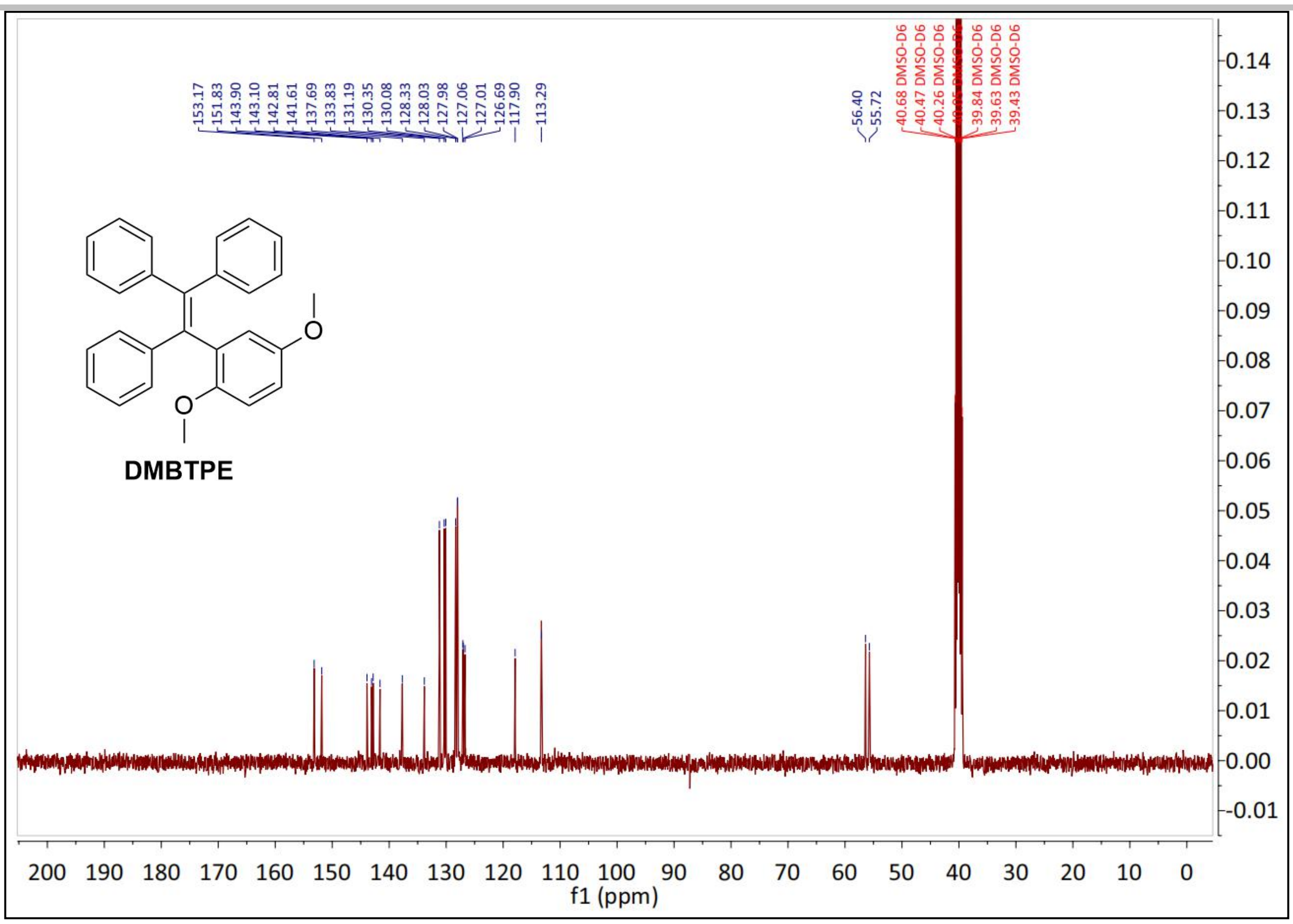

Figure S2. ^{13}C NMR spectrum of **DMBTPE** in DMSO-d_6.^{13}C NMR (101 MHz, DMSO-d6): δ153.17, 151.83, 143.89, 143.10, 142.81, 141.61, 137.69, 133.83, 131.18, 130.35, 130.08, 128.33, 128.03, 127.98, 127.06, 127.01, 126.69, 117.90, 113.30, 56.40, 55.72.

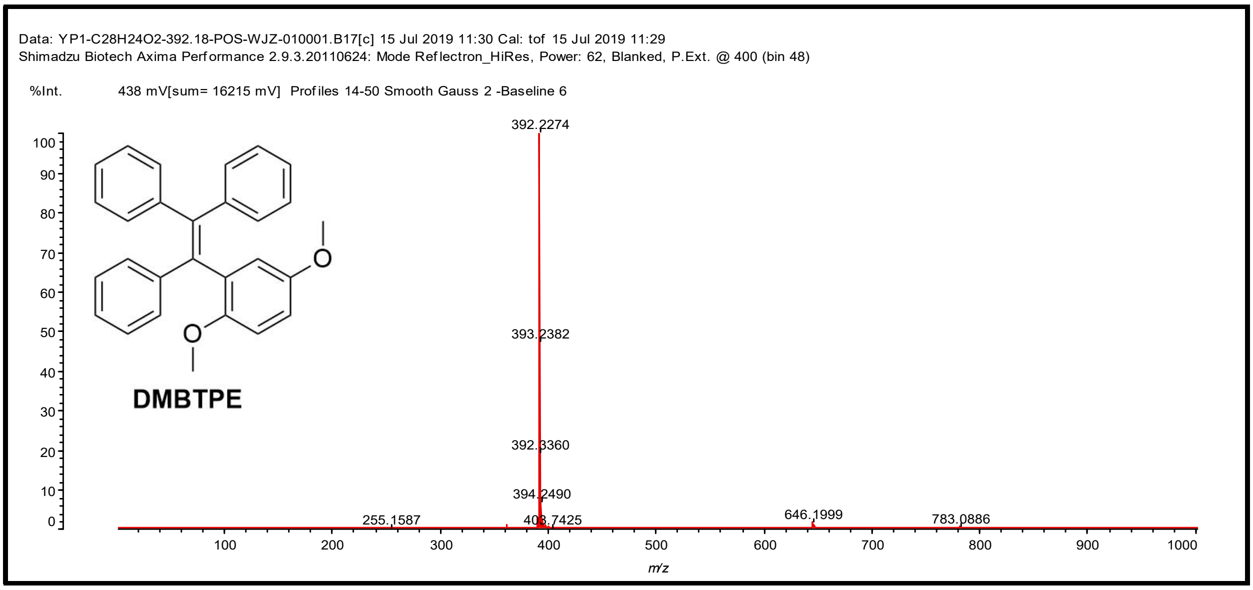

Figure S3. Mass spectrum of **DMBTPE**. Calcd for $C_{28}H_{24}O_2$ $[M]^+$, 392.18; found, 392.22.

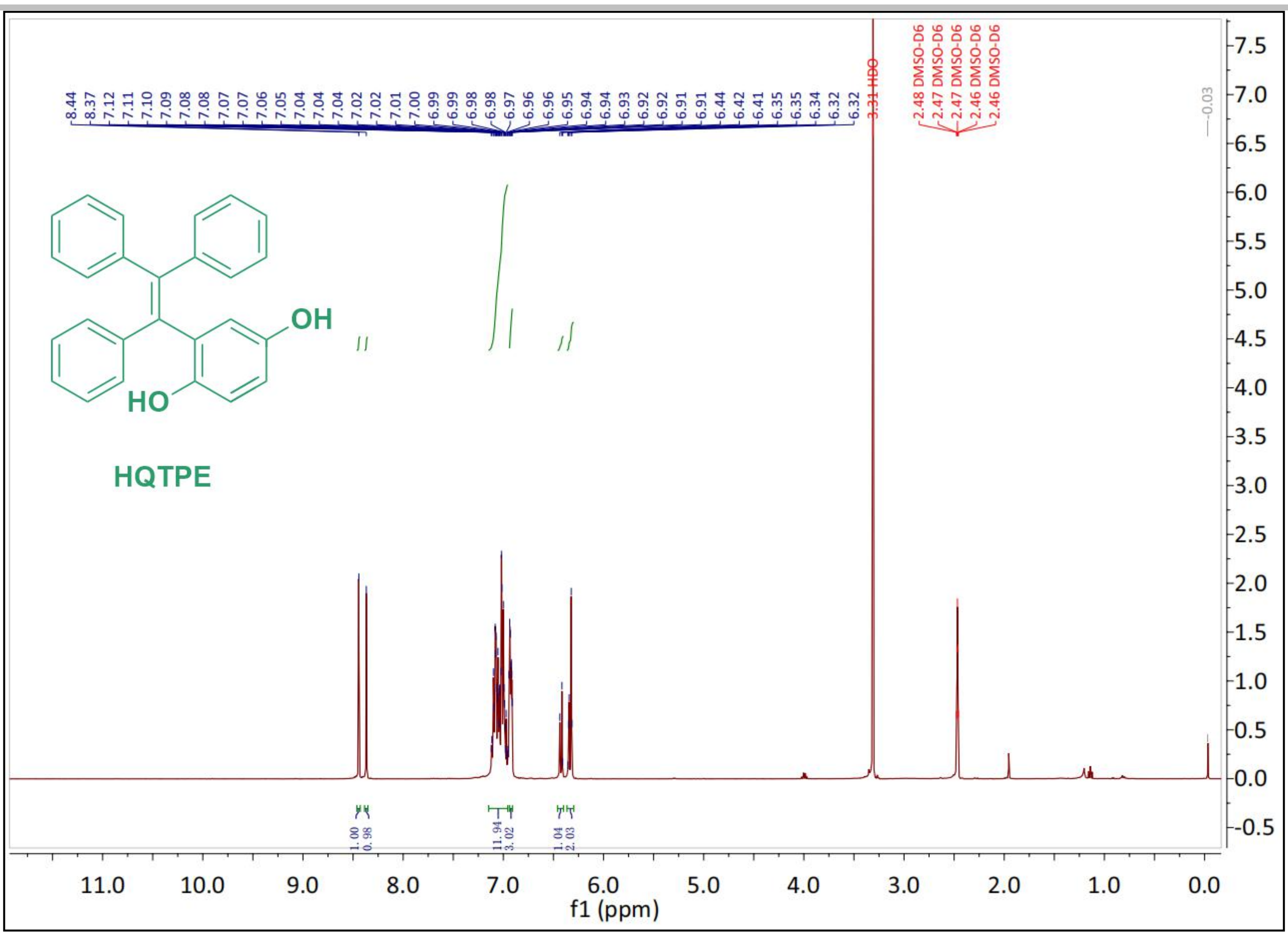

Figure S4. ^{1}H NMR spectrum of **HQTPE** in DMSO-d_6. ^{1}H NMR (400 MHz, DMSO-d_6): δ8.44 (s, 1H, of phenolic hydroxyl), 8.37 (s, 1H, of phenolic hydroxyl), 7.15-6.96 (m, 12H, of three benzene rings), 6.94-6.91 (m, 3H, of three benzene rings), 6.43 (d, J = 8.2 Hz, 1H, of the hydroquinone ring), 6.36-6.30 (m, 2H, of the hydroquinone ring).

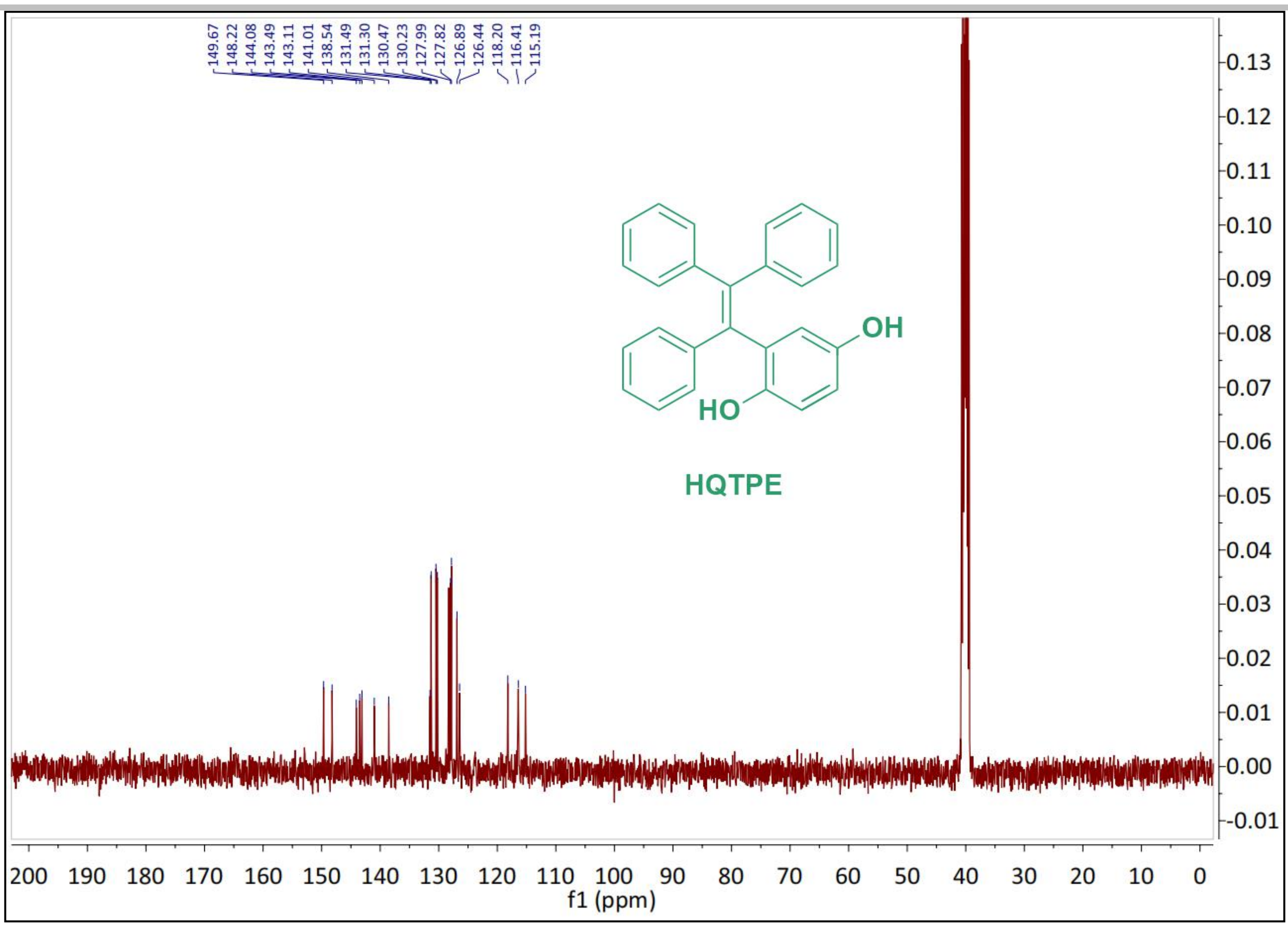

Figure S5. ^{13}C NMR spectrum of **HQTPE** in DMSO-d_6. ^{13}C NMR (101 MHz, DMSO-d_6): δ149.67, 148.22, 144.08, 143.49, 143.11, 141.01, 138.54, 131.49, 131.30, 130.47, 130.23, 127.99, 127.82, 126.89, 126.44, 118.20, 116.41, 115.19.

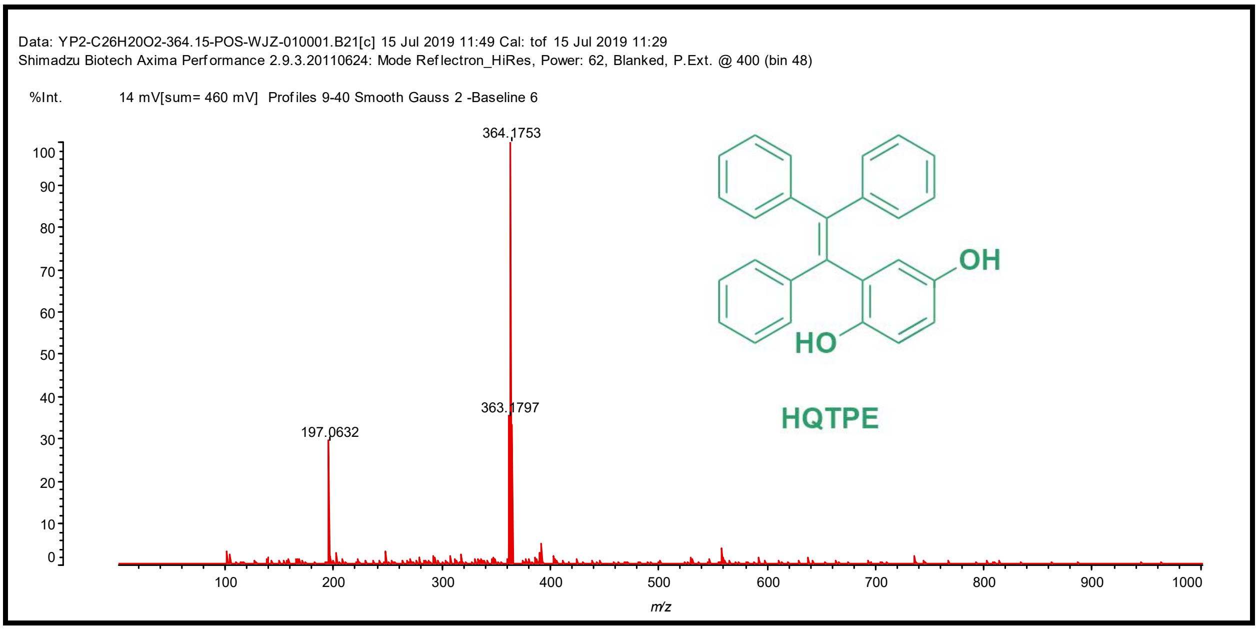

Figure S6. Mass spectrum of **HQTPE**. Calcd for $C_{26}H_{20}O_2$ $[M]^+$, 364.15; found, 364.18.

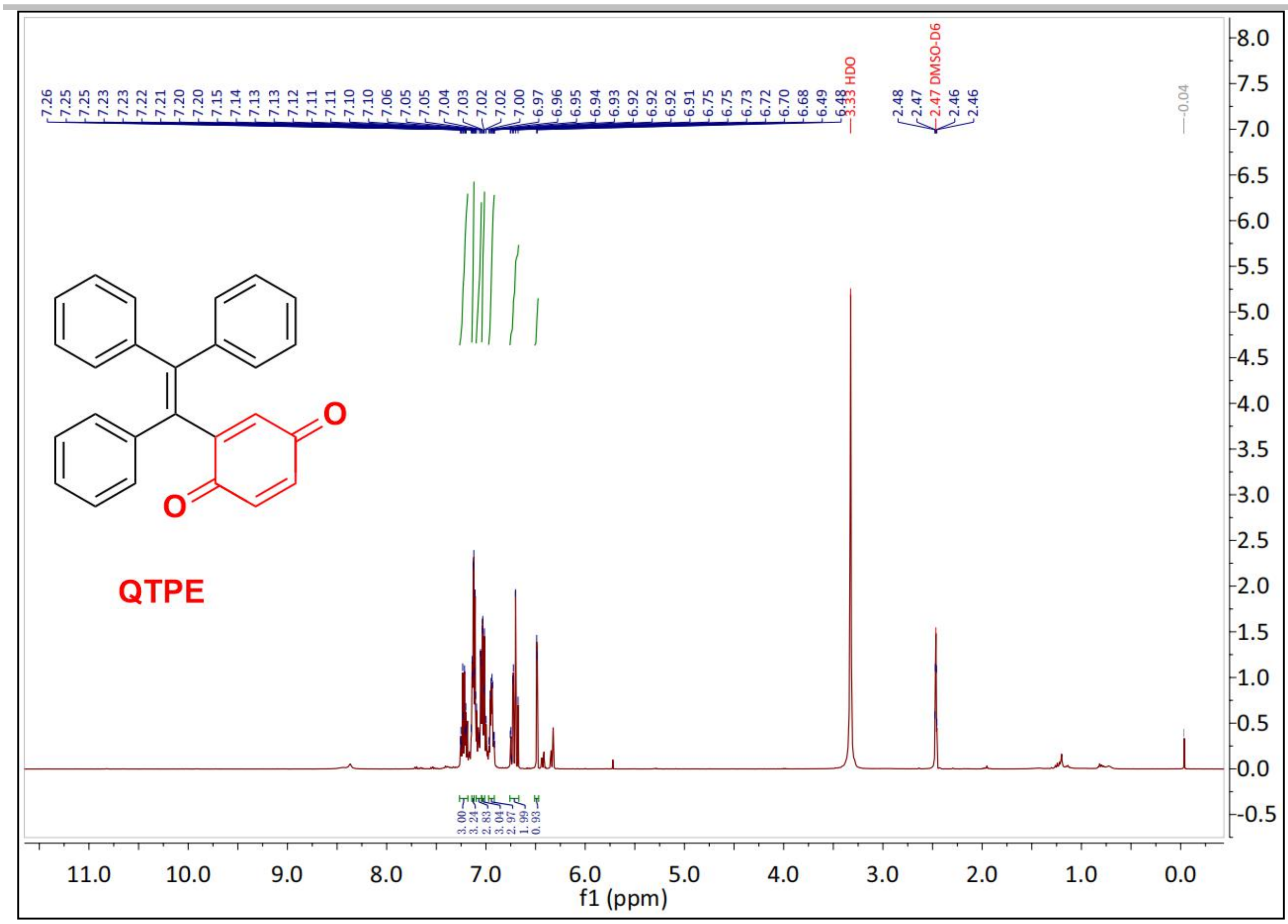

Figure S7. ^{1}H NMR spectrum of **QTPE** in DMSO-d_6.^{1}H NMR (400 MHz, DMSO-d_6): δ7.27- 7.18 (m, 3H, of three benzene rings), 7.14-7.12 (m, 3H, of three benzene rings), 7.10-7.04 (m, 3H, of three benzene rings), 7.04-7.01 (m, 3H, of three benzene rings), 6.94 (tt, J = 5.8, 3.4 Hz, 3H, of three benzene rings), 6.76-6.67 (m, 2H, of the benzoquinone ring), 6.49 (d, J = 2.3 Hz, 1H, of the benzoquinone ring).

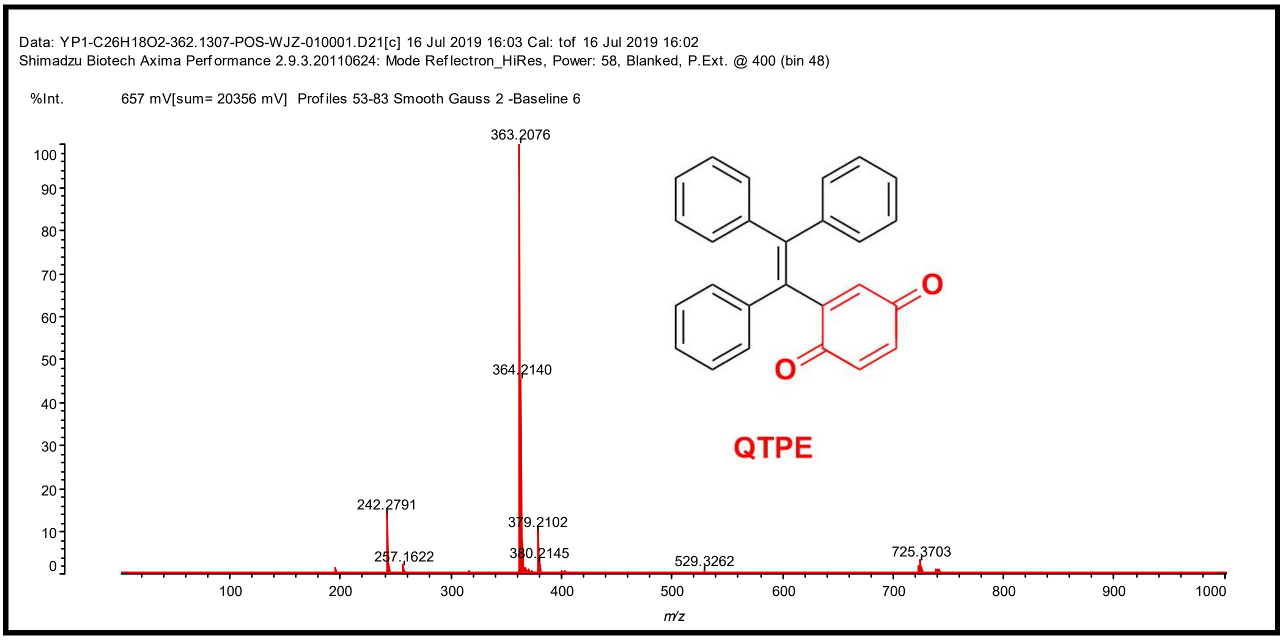

Figure S8. Mass spectrum of **QTPE**. Calcd for $C_{26}H_{18}O_2$ $[M+H]^+$, 363.14; found, 363.21.

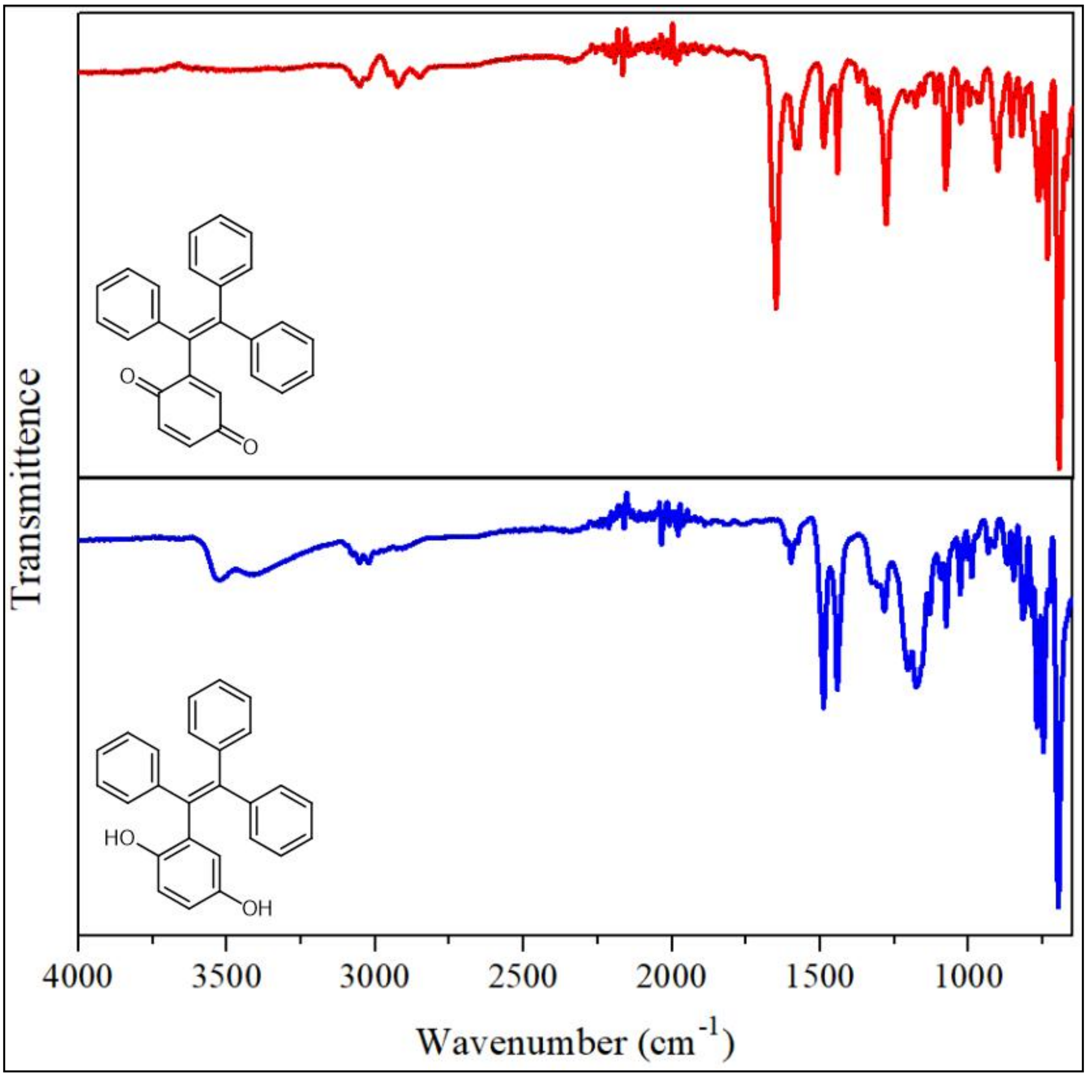

Figure S9. FT-IR spectra of **HQTPE** and **QTPE**. **HQTPE**: ν(OH) 3524 (m) and 3409 (m), ν(arC-H) 1176 (s), 770 (s), 748 (s) and 697 (vs), ν(arC-C) 1489 (s) and 1443 (s) cm^{-1}. **QTPE**: ν(C=O) 1651 (m), ν(arC-H) 1080 (s), 768 (s), 736 (s) and 697 (vs), ν(arC-C) 1444 (s) and 1281 (s) cm^{-1}.

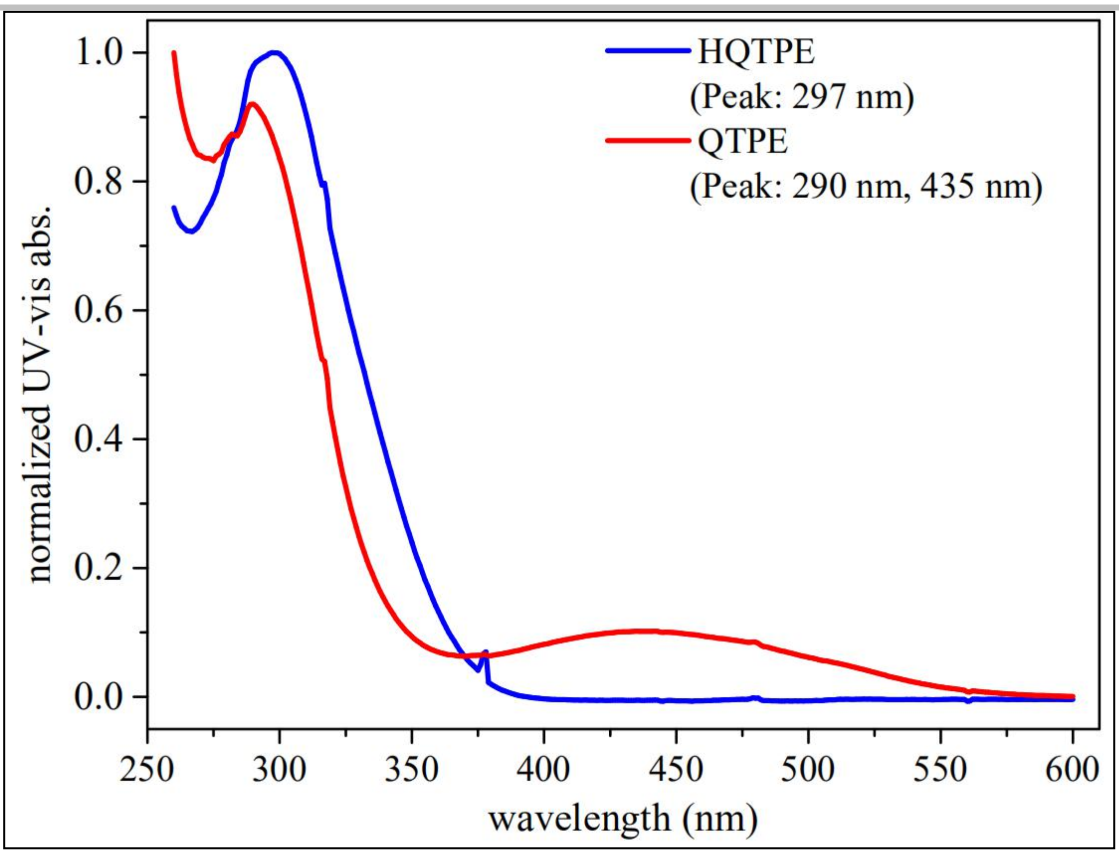

Figure S10. UV-vis absorption spectra of **HQTPE** and **QTPE**.

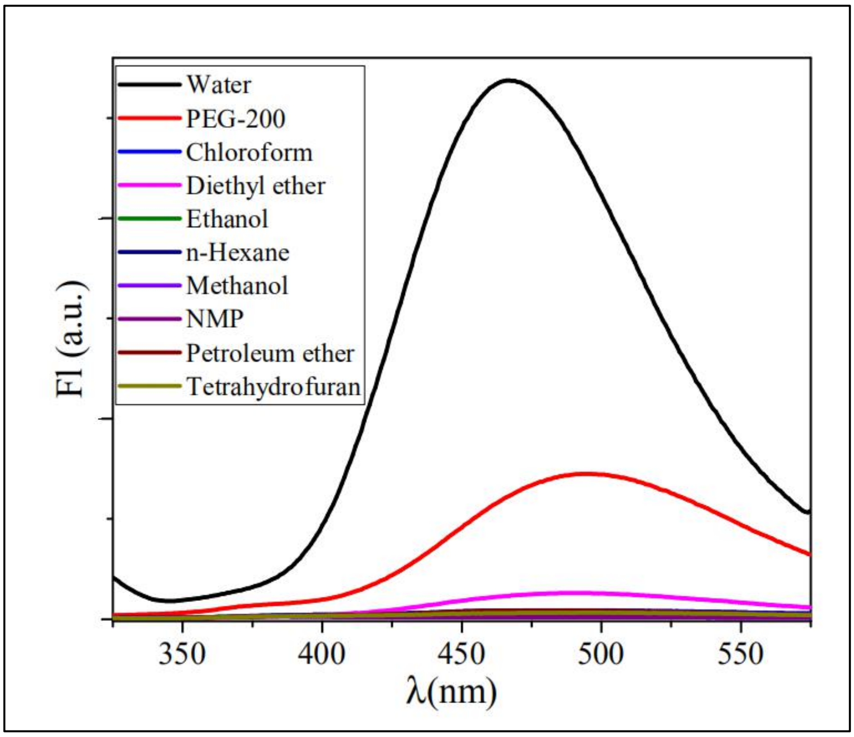

Figure S11. Fluorescence spectra of 100 μM **HQTPE** in different solvants (water, chloroform, methanol, ethanol, tetrahydrofuran, N-methylpyrrolidone, PEG-200, petroleum ether, n-hexane).

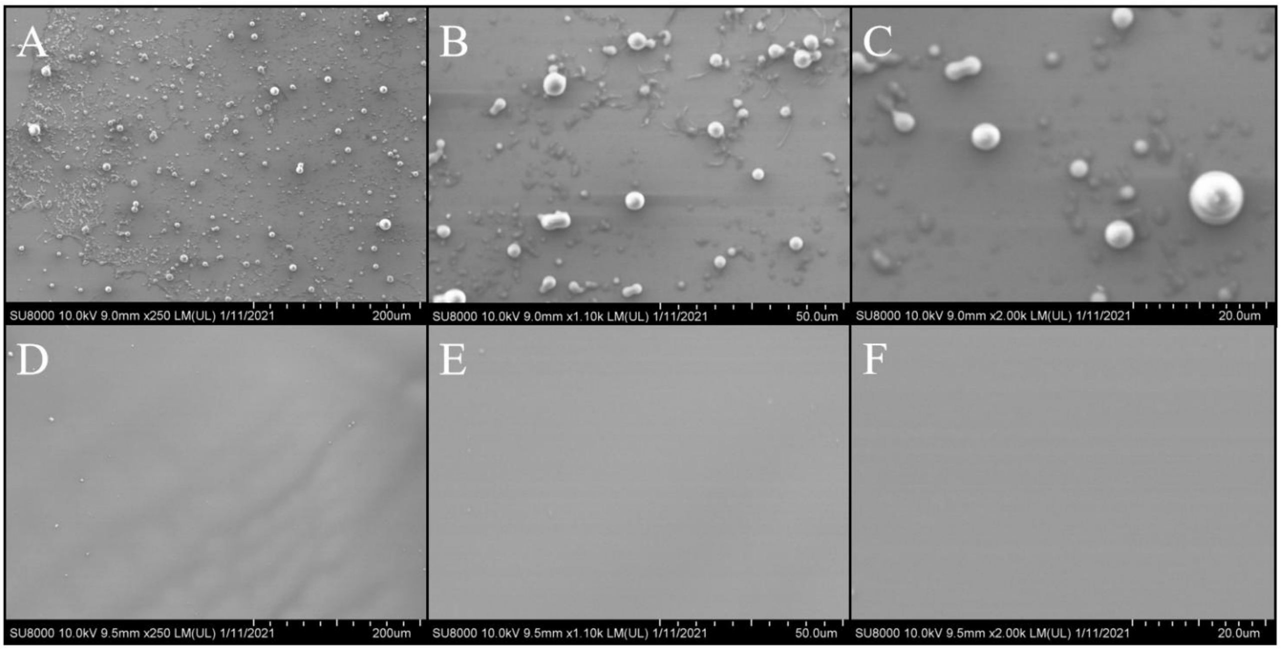

Figure S12. Scanning electron micrographs of **HQTPE** particles, precipitated from (A,B,C) the 99 vol% water/THF solvent and (D,E,F) the pure THF.

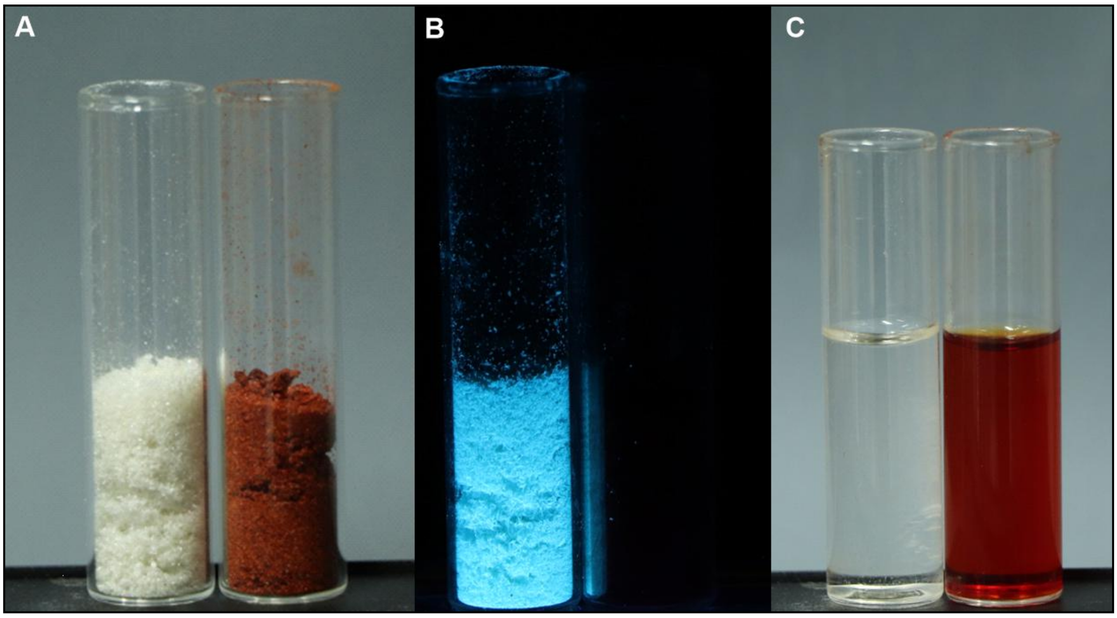

Figure S13. Photographs of **HQTPE/QTPE** solid (A) under white light, and (B) excited by UV light. (C) Photographs of **HQTPE/QTPE** solutions under white light.

Application

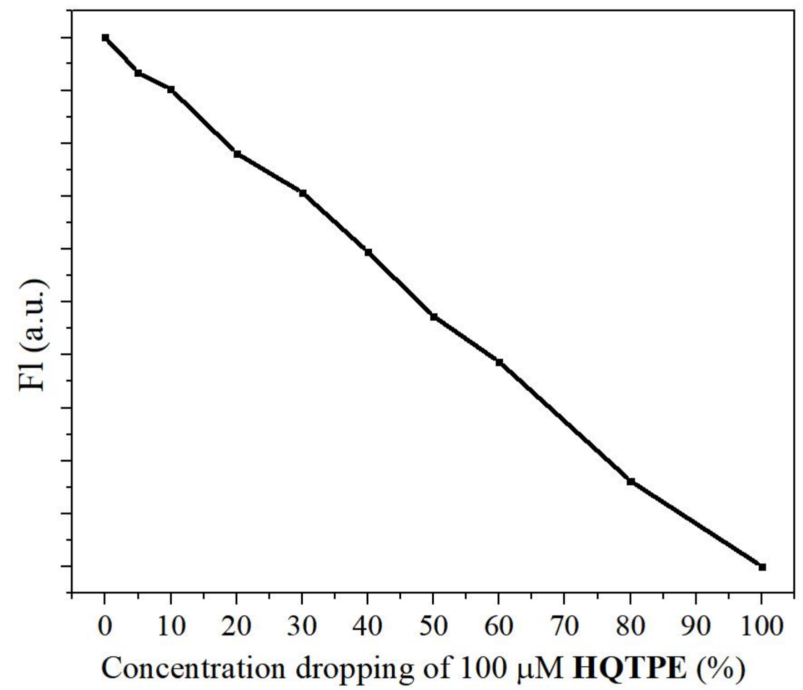

Figure S14. Fitting curve of the FL intensity of **HQTPE** solutions at different concentrations without the oxidated specie **QTPE**.

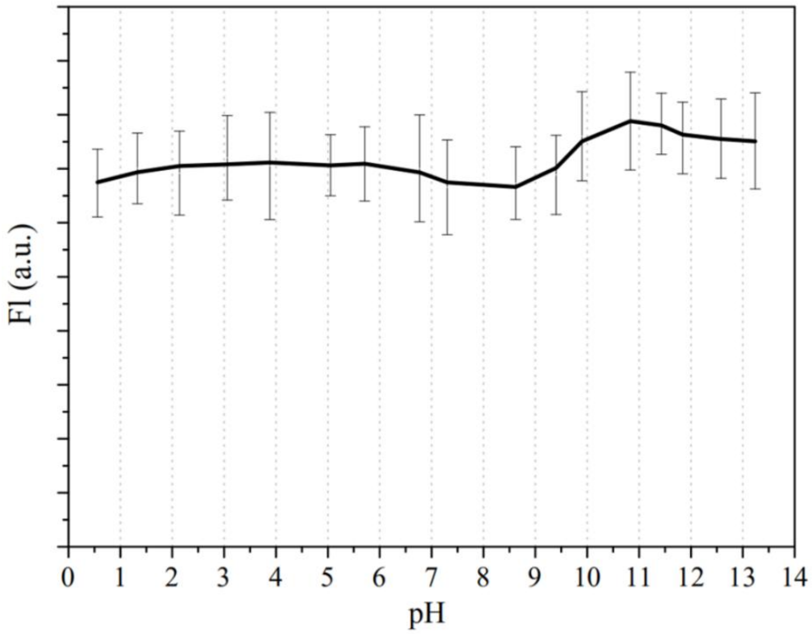

Figure S15. Fitting curve of the FL intensity of **DMBTPE** solutions under different pH conditions. Error bars show standard deviations of three independent measurements.

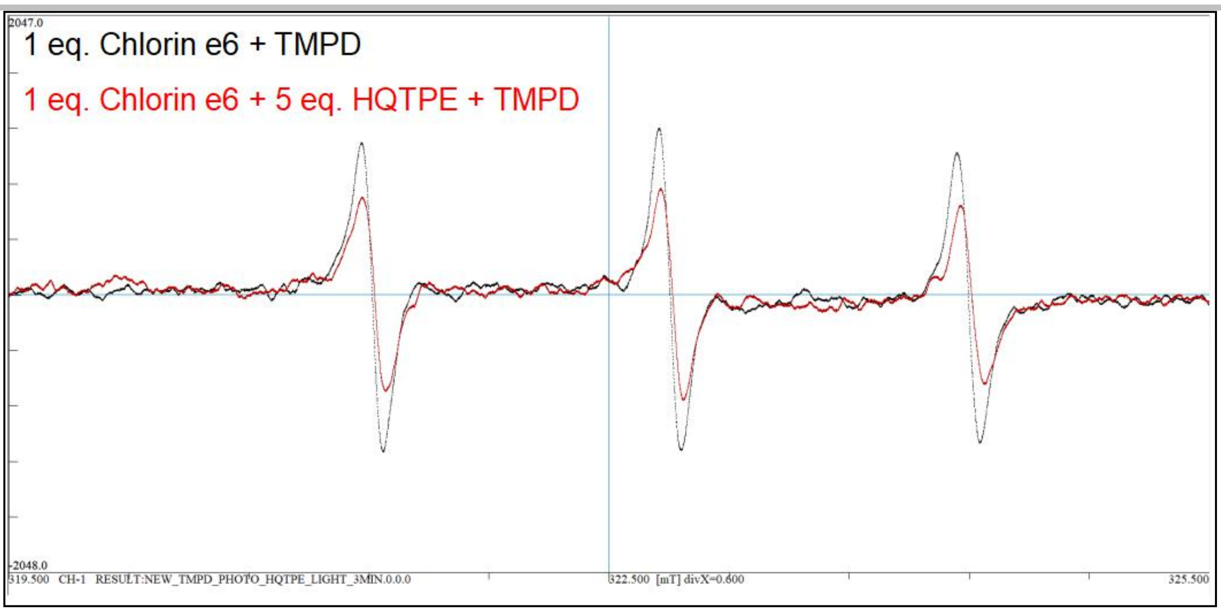

Figure S16. Influence of **HQTPE** on the capture of singlet oxygen ($^1O_2$) generated from Chlorin e6: EPR spectra of Chrorin e6 (black) and mixed with **HQTPE** as the singlet oxygen neutralizer (red) in THF solution, where chlorin e6 was used as the photosensitizer and 2,2,6,6-tetramethyl-4-piperidone (TMPD) was used as the radical trapping agent to show EPR signals. After adding **HQTPE**, the signal intensity had a significant decrease, which demonstrated that $^1O_2$ generated from Chlorin e6 could be efficiently consumed by **HQTPE**.

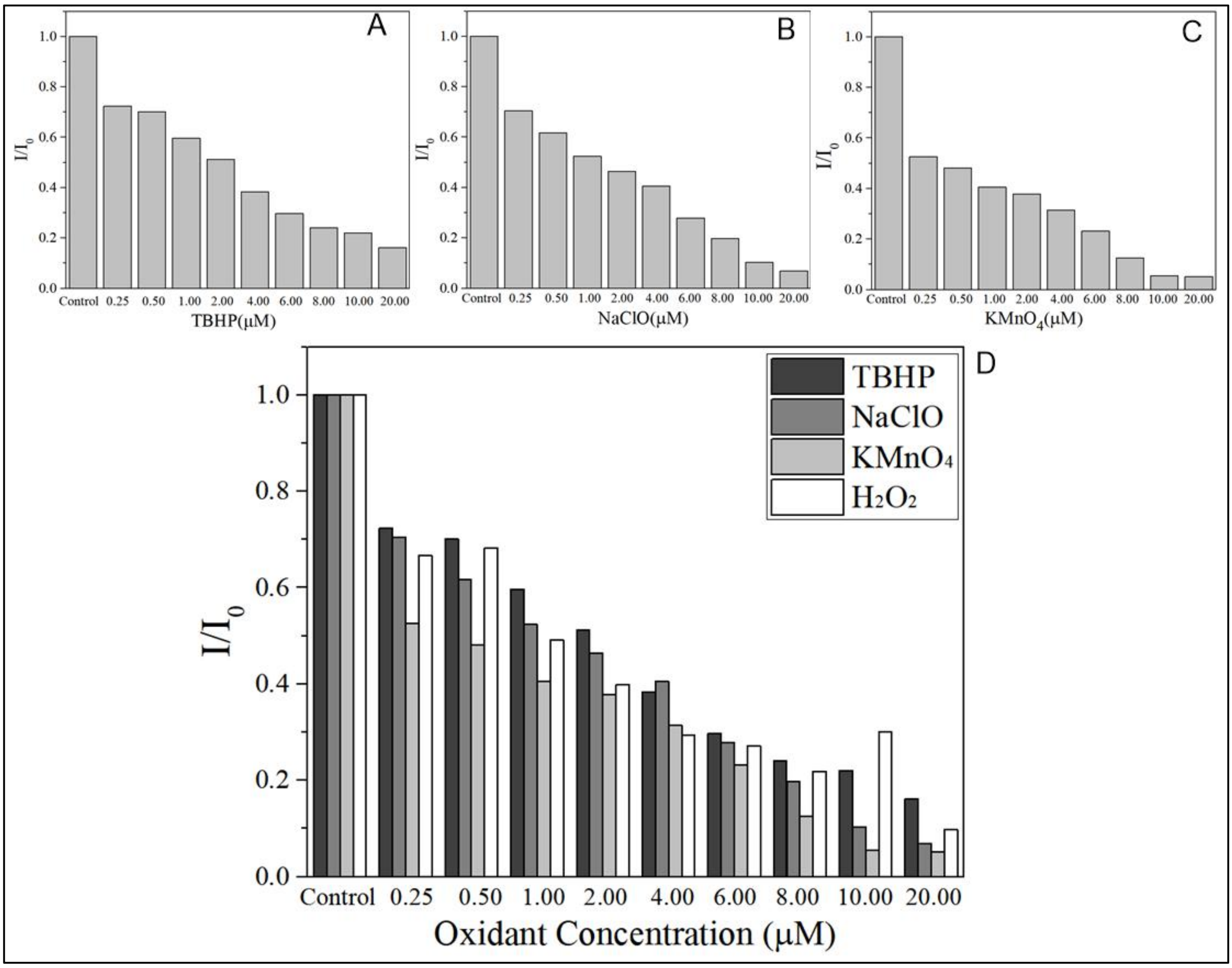

Figure S17. **Efficient response of HQTPE to different oxidants.** (A) Relative fluorescence intensity at 462 nm of 10 μM **HQTPE** in water before and after addition of TBHP, or (B) NaClO, or (C) $KMnO_4$ at various concentrations. (D) Columnar comparison of the relative fluorescence intensities of different oxidants tested. I_0 is the fluorescence intensity of 10 μM **HQTPE** in water at 462 nm (control) and I is the corresponding fluorescence in the presence of testing species with gradient concentrations.

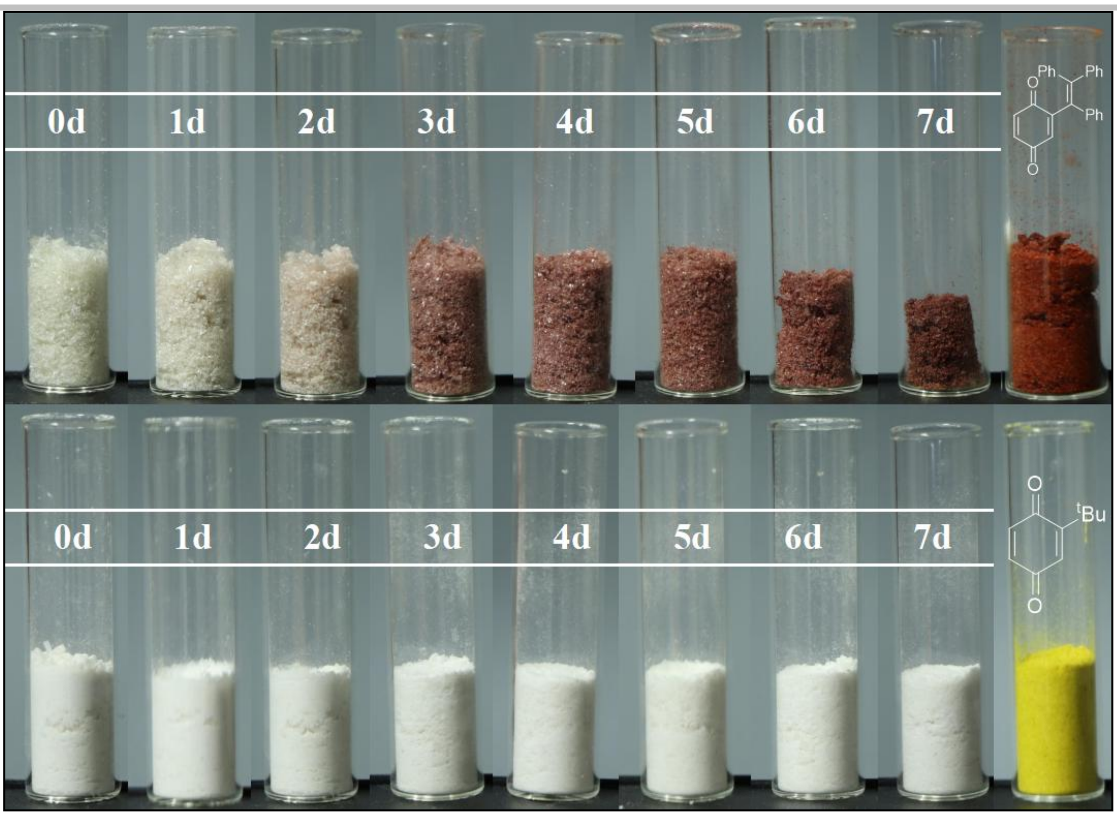

Figure S18. Photographs of **HQTPE** and **TBHQ** powder exposed to air within seven days under white light. We could find **HQTPE** possesses better deoxygenation activity than **TBHQ** by the comparison of their oxidized state.

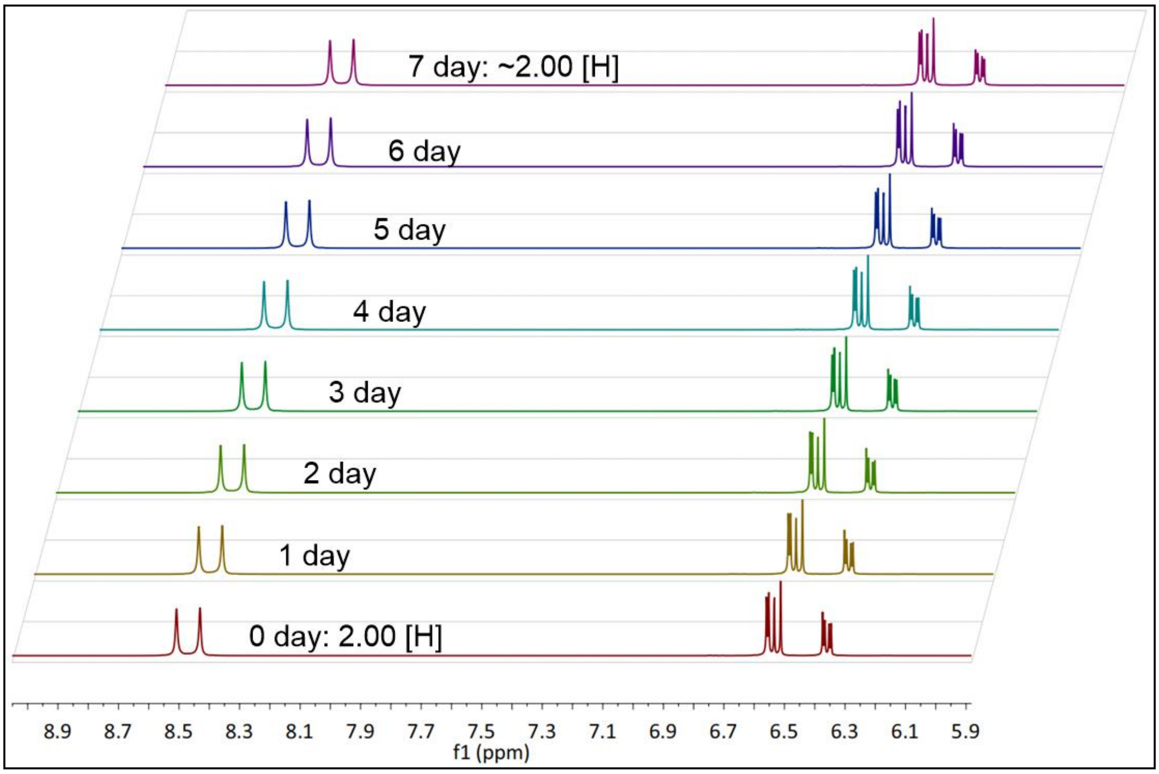

Figure S19. ^{1}H NMR spectra in DMSO-d_6 of **TBHQ** solution. No obvious changes could be found after the week-long exposure to air.

Computational Studies

Supplementary Note 2

Unless otherwise specified, all DFT calculations in SI were also performed with the methods described in the main article.

For corresponding mono-deprotonated anion of **HQTPE**, we have performed ground state energy calculation of two possible structures beforehand (Fig. S20), and found the meta-anion has lower ground state free energy (ΔG=-2.9 kcal mol^{-1}) and higher stability. So we used the more stable structure when studying the properties of mono-deprotonated anion **HQTPE** at latter studies.

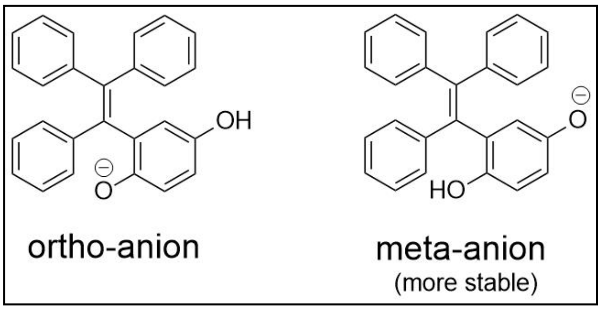

Figure S20. Two possible structures of mono-deprotonated anion. The proton in meta position could dissociate more easily.

When analyzing the frontier molecule orbitals (FMO), all energy levels of FMO showed were calculated from the optimized S_1 geometry using TD-DFT method and cpcm solution model with THF as the solvent.

When analyzing the change of Gibbs free energy in the excited state electron transfer process, all the geometry optimizations, including neutrals, cations and anions, were performed in the gas phase with the B3LYP-D3 functional and 6-31G(d) basis and Gibbs free energies were calculated with B3LYP-D3 functional and 6-311+G(d,p) basis, using the cpcm solvation model with THF as the solvent for comparison.

Natural transition orbital analysis was performed with Multiwfn.[4]

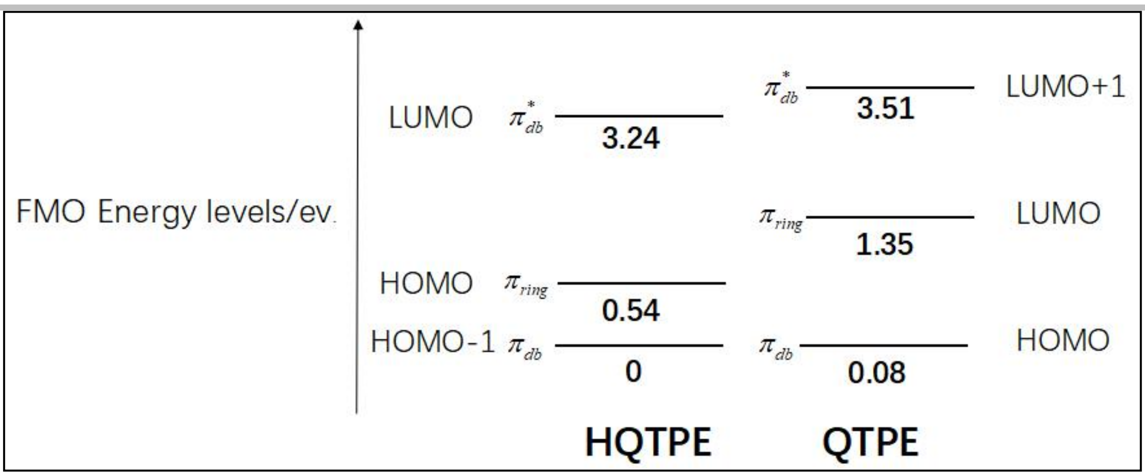

Figure S21. The calculated FMO energy levels of **HQTPE** and **QTPE**, π means these orbitals are all π orbitals, subscript 'db' (double-bond) means corresponding orbitals distribute mainly on the double bond area, subscript 'ring' means corresponding orbitals distribute mainly on the phenol aromatic ring.

Table S1. **The contribution percent of transitions between noted FMOs to S_0-S_1 transition in HQTPE and QTPE**, calculated through TD-DFT**.**

Contribution percentage	HQTPE	QTPE
HOMO ->LUMO+1	-	-
HOMO -> LUMO	68.6%	99.5%
HOMO-1 -> LUMO	29.7%	-

In Fig. S21, we have listed the energy levels of frontier molecule orbitals related to S_0-S_1 transition of **HQTPE** and **QTPE**. On **HQTPE** case, narrow energy gap between HOMO-1 and HOMO allow local excitation (LE) transition between HOMO-1 and LUMO to mix into S_0-S_1 transition which is dominant by charge transfer (CT) transition between HOMO and LUMO, therefore contribute to the stronger oscillator strength. On **QTPE** case, though the three FMO still have similar density distribution respectively, the energy gap between them changed, and HOMO-1 and HOMO, which have the two lower energy in **HQTPE**, became new HOMO and LUMO in **QTPE** since total electron number decreased two. These altogether may result in S_0-S_1 transition of **QTPE** a pure CT transition dominant by electron transition between HOMO mainly distributed on the double bond and LUMO mainly distributed on the benzoquinone ring with weak oscillator strength.

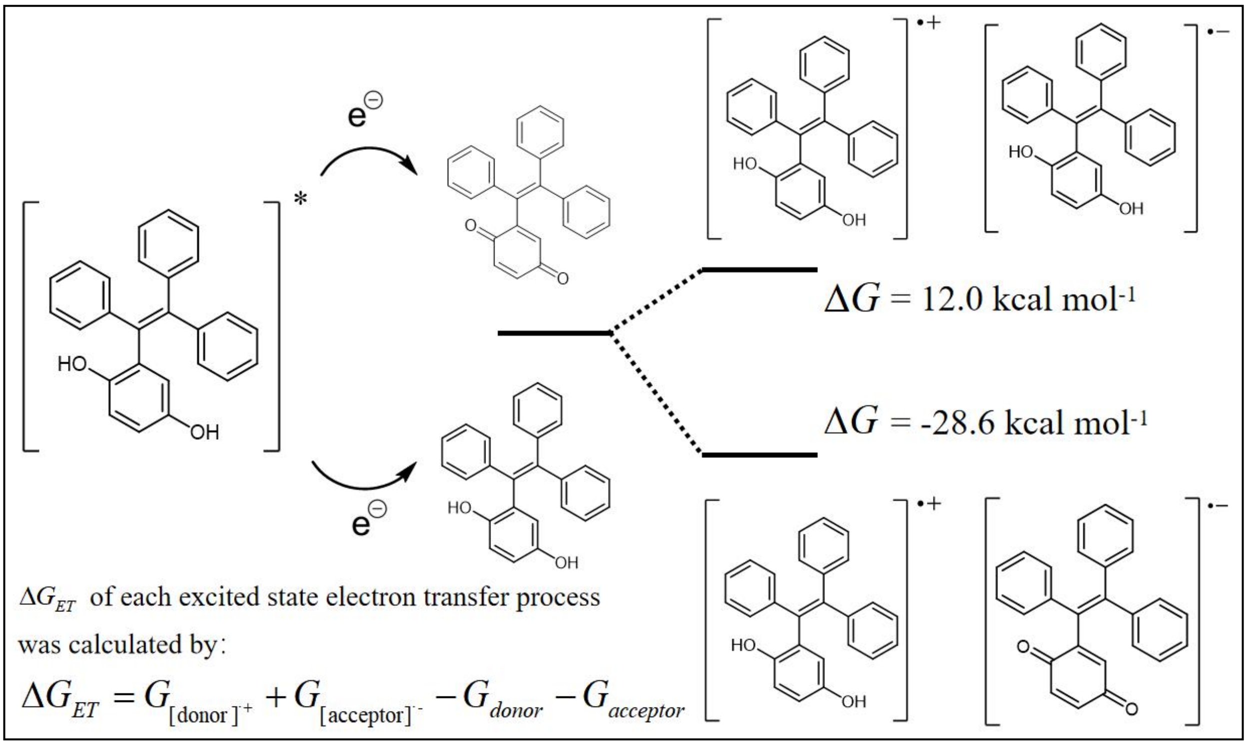

Figure S22 Proposed excited state electron transfer pathway of excited-state **HQTPE**.

For nonlinear fluorescence quenching process, it has been confirmed the molecules with quinone moiety can quench fluorescence. The DFT calculations also indicate that the endergonic electron transfer (ET) from excited-state **HQTPE** to another ground state **HQTPE** molecule will be highly exergonic if **QTPE** added and acting as the electron acceptor instead. Therefore, according to the Marcus theory,[5] the excited state electron transfer process will be significantly accelerated and compete with the radiation process to relax the excited state.

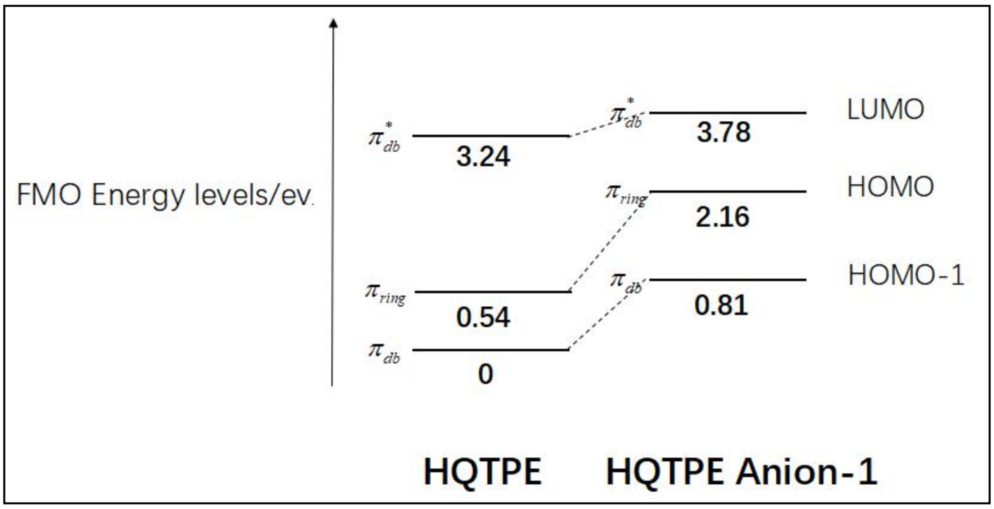

Figure S23. The calculated FMO energy levels of **HQTPE** and its mono-deprotonated anion (solution phase), π means these orbitals are all π orbitals, subscript 'db' (double-bond) means corresponding orbitals distribute mainly on the double bond area, subscript 'ring' means corresponding orbital distribute mainly on the phenol aromatic ring.

Table S2. **The contribution percent of transitions between noted FMOs to S_0-S_1 transition in HQTPE and HQTPE Anion-1**, calculated through TD-DFT.

**Contribution percentage**	**HQTPE**	**HQTPE Anion-1**
HOMO ->LUMO+1	-	-
HOMO -> LUMO	68.6%	99.5%
HOMO-1 -> LUMO	29.7%	-

To find the reason of overlap absence, we analyzed the frontier molecular orbitals (FMO) of **HQTPE** and its mono-deprotonated anion. We found that because of the higher electron density in the phenol ring of the anion, the HOMO energy significantly increased, enlarging the gap between HOMO-1 (π_{db}) and HOMO (π_{ring}). We assumed this larger gap can make it more difficult for local excitation (LE) transition between HOMO-1 and LUMO to involve in the S_0-S_1 transition, resulting in the S_0-S_1 transition of the anion as a purer CT transition with weaker oscillator strength.

Table S3. **Some Photochemical properties calculated on S_1 state of the relative compounds**. E_{ad} denotes the adiabatic excitation energy E_{vert} denotes the vertical excitation energy at optimized S_1 geometry and *f* denotes the oscillator strength of S_0-S_1 transition. CI coefficients denotes the configuration interaction coefficients corresponding to S_0-S_1 transition.

Compound	E_{ad} / eV	E_{vert} / eV	*f*	CI coefficients
**HQTPE**	2.98	2.44	0.1635	HOMO->LUMO 0.586<br>HOMO-1->LUMO -0.385
**QTPE**	1.34	0.65	0.0028	HOMO->LUMO 0.705
ANION-1 (mono-deprotonated)	1.38	0.95	0.0064	HOMO->LUMO 0.706
ANION-2 (di-deprotonated)	0.66	0.10	0.0002	HOMO->LUMO 0.706

Supplementary References

1. Zhang, X. *et al.* Piezofluorochromism of an aggregation-induced emission compound derived from tetraphenylethylene. *Chem. - An Asian J.* **6**, 808–811 (2011).
2. Lucas, N., Hook, J., McDonagh, A. & Colbran, S. Titanium Dioxide Nanoparticles Functionalized with Pd and W Complexes of a Catecholphosphane Ligand. *Eur. J. Inorg. Chem.* **2005**, 496–503 (2005).
3. Daumas, M., Vo-Quang, Y., Vo-Quang, L. & Le Goffic, F. A new and efficient heterogeneous system for the oxidative cleavage of 1,2-diols and the oxidation of Hydroquinones. *Synth.* **1989**, 64–65 (1989).
4. Lu, T. & Chen, F. Multiwfn: A multifunctional wavefunction analyzer. *J. Comput. Chem.* **33**, 580–592 (2012).
5. Marcus, R. A. On the theory of oxidation-reduction reactions involving electron transfer. I. *J. Chem. Phys.* **24**, 966–978 (1956).
