# Supplementary material for: Intrinsic hydroquinone-functionalized aggregation-induced emission core shows redox and pH sensitivity
Source: Commun Chem. 2021 Apr 23;4:55. doi: 10.1038/s42004-021-00492-4 (PMC9814920; doi:10.1038/s42004-021-00492-4)
Supplement: Supplementary file 3 — Description of Additional Supplementary Files [file 42004_2021_492_MOESM3_ESM.pdf]

Description of Additional Supplementary Files

File Name: Supplementary Data 1

Description: Contains the cartesian coordinates for each related compound or intermediate, including HQTPE, QTPE and the corresponding anionic species.
