# Supplementary material for: Intrinsic hydroquinone-functionalized aggregation-induced emission core shows redox and pH sensitivity
Source: Commun Chem. 2021 Apr 23;4:55. doi: 10.1038/s42004-021-00492-4 (PMC9814920; doi:10.1038/s42004-021-00492-4)
Supplement: Supplementary file 4 — Supplementary Data 1 [file 42004_2021_492_MOESM4_ESM.zip › Supplementary Data 1.doc]

**Cartesian Coordinates**

**HQTPE**

Optimized ground state geometry

SCF energy = -1153.679546 a.u.

C	-0.08271 -0.53744 -0.05228

C	-0.46589	0.76915 -0.05222

C	0.51641	1.8677	-0.25762

C	1.44853	1.8161	-1.30584

C	0.51042	2.99431	0.57952

C	2.36682	2.8463	-1.49196

H	1.44145	0.9615	-1.97437

C	1.43449	4.02147	0.39863

H	-0.22272	3.05524	1.3785

C	2.36855	3.94994 -0.63647

H	3.08012	2.78976 -2.30964

H	1.42329	4.88029	1.06436

H	3.08625	4.7526	-0.78135

C	-1.87307	1.1997	0.1648

C	-2.6445	0.66509	1.20873

C	-2.44494	2.18917 -0.65051

C	-3.95486	1.0882	1.41474

H	-2.20864 -0.09043	1.85376

C	-3.75941	2.60616 -0.45086

H	-1.85051	2.62502 -1.44786

C	-4.52032	2.05617	0.58208

H	-4.5352	0.66338	2.22916

H	-4.18879	3.3639	-1.1006

H	-5.54352	2.38452	0.74198

C	-1.04074 -1.67394 -0.07098

C	-0.83274 -2.78307	0.76239

C	-2.14379 -1.6856	-0.93861

C	-1.72059 -3.85766	0.75505

H	0.02962 -2.79469	1.4224

C	-3.02636 -2.76095 -0.95163

H	-2.29986 -0.84474 -1.60544

C	-2.82271 -3.85	-0.10042

H	-1.54925 -4.70258	1.41667

H	-3.87313 -2.75287 -1.63254

H	-3.51343 -4.68864 -0.11064

C	1.35422 -0.92037	0.02229

C	1.94224 -1.73396 -0.95962

C	2.13942 -0.50933	1.10592

C	3.2771	-2.1239	-0.84

C	3.47606 -0.89027	1.22093

H	1.68528	0.12832	1.8604

C	4.04567 -1.71016	0.24535

H	3.72632 -2.74736 -1.61075

H	5.08422 -2.00807	0.34196

O	1.1702	-2.07102 -2.0431

H	1.66582 -2.69296 -2.59711

O	4.26942 -0.50176	2.2706

H	3.76179	0.10323	2.83269

**HQTPE**

Optimized S_1_ excited state geometry

SCF energy = -1153.570911 a.u.

C	-0.18486 -0.57456 -0.08721

C	-0.56038	0.80185 -0.09122

C	0.428	1.78126 -0.525

C	1.37952	1.45163 -1.52631

C	0.56535	3.0507	0.09423

C	2.42779	2.31054 -1.84668

H	1.23725	0.53426 -2.08792

C	1.61013	3.90207 -0.2302

H	-0.14705	3.33598	0.86221

C	2.56265	3.53752 -1.19672

H	3.13306	2.02894 -2.62579

H	1.69802	4.85887	0.27932

H	3.3796	4.20905 -1.44477

C	-1.88139	1.24932	0.34021

C	-2.6225	0.5316	1.31176

C	-2.484	2.41102 -0.19896

C	-3.87478	0.96286	1.72788

H	-2.1909	-0.36759	1.73738

C	-3.73689	2.84075	0.22317

H	-1.95784	2.96414 -0.9706

C	-4.44625	2.12142	1.19054

H	-4.41284	0.39162	2.48059

H	-4.17113	3.73581 -0.21574

H	-5.42942	2.45218	1.51328

C	-1.07299 -1.70041 -0.26915

C	-0.76522 -2.99099	0.23844

C	-2.30771 -1.573	-0.96294

C	-1.61995 -4.07125	0.06063

H	0.1501	-3.13607	0.8074

C	-3.15843 -2.65516 -1.12952

H	-2.57517 -0.60968 -1.38137

C	-2.83048 -3.91946 -0.62353

H	-1.34674 -5.04017	0.47317

H	-4.09036 -2.51521 -1.67254

H	-3.50216 -4.76229 -0.75695

C	1.24372 -0.86717	0.19788

C	2.08316 -1.64854 -0.67635

C	1.8514	-0.37908	1.3363

C	3.45553 -1.86951 -0.40257

C	3.225	-0.57039	1.59496

H	1.25328	0.22187	2.01474

C	4.0363	-1.33754	0.72394

H	4.04783 -2.45684 -1.09971

H	5.08578 -1.47356	0.95504

O	1.51574 -2.141	-1.77669

H	2.1674	-2.62877 -2.30927

O	3.82819 -0.04051	2.66761

H	3.19071	0.48897	3.17754
