# Supplementary material for: Intrinsic hydroquinone-functionalized aggregation-induced emission core shows redox and pH sensitivity
Source: Commun Chem. 2021 Apr 23;4:55. doi: 10.1038/s42004-021-00492-4 (PMC9814920; doi:10.1038/s42004-021-00492-4)
Supplement: Supplementary file 4 — Supplementary Data 1 [file 42004_2021_492_MOESM4_ESM.zip › Supplementary Data 2.doc]

**Cartesian Coordinates**

**QTPE**

Optimized ground state geometry

SCF energy = -1152.436827 a.u.

C	0.02496 -0.51985 -0.00144

C	0.52487	0.7459	-0.01134

C	-0.36337	1.92328	0.17522

C	-1.27991	1.9644	1.23736

C	-0.28225	3.02467 -0.69006

C	-2.11736	3.0645	1.40925

H	-1.31693	1.13261	1.93414

C	-1.12697	4.11968 -0.52403

H	0.44011	3.00966 -1.50075

C	-2.04924	4.14206	0.52455

H	-2.81865	3.08322	2.23881

H	-1.06529	4.95749 -1.21283

H	-2.70397	4.99873	0.6564

C	1.96958	1.04109 -0.18745

C	2.72908	0.39152 -1.17388

C	2.5941	2.01401	0.60928

C	4.07783	0.69078 -1.34317

H	2.2529	-0.35011 -1.80603

C	3.94695	2.30487	0.44739

H	2.01119	2.53507	1.36246

C	4.69385	1.64375 -0.52899

H	4.64884	0.18149 -2.11433

H	4.41718	3.0511	1.08164

H	5.74724	1.87464 -0.66008

C	0.85603 -1.74893	0.07759

C	0.58064 -2.84162 -0.75762

C	1.89867 -1.85981	1.01068

C	1.34673 -4.00477 -0.68456

H	-0.23087 -2.77202 -1.47639

C	2.65963 -3.02113	1.08603

H	2.10064 -1.02907	1.67767

C	2.39007 -4.09738	0.23581

H	1.12629 -4.83772 -1.34636

H	3.46118 -3.09177	1.81588

H	2.98538 -5.00395	0.29752

C	-1.43295 -0.77524 -0.09458

C	-2.09274 -1.48724	1.04862

C	-2.16987 -0.41612 -1.16776

C	-3.52216 -1.85588	0.90323

C	-3.60685 -0.73005 -1.2851

H	-1.73146	0.12734 -1.99898

C	-4.23306 -1.49352 -0.17499

H	-3.95142 -2.40444	1.73657

H	-5.28682 -1.72856 -0.29332

O	-1.49245 -1.70962	2.09249

O	-4.26132 -0.39318 -2.26685

**QTPE**

Optimized S_1_ excited state geometry

SCF energy = -1152.387439 a.u.

C	-0.04048 -0.57431	0.05307

C	0.47431	0.73721	0.14915

C	-0.35884	1.89856	0.48463

C	-1.23284	1.87795	1.58914

C	-0.24368	3.08085 -0.2781

C	-1.97279	3.00949	1.90617

H	-1.32153	0.969	2.17386

C	-1.00216	4.20275	0.03666

H	0.41454	3.09532 -1.14069

C	-1.86505	4.17133	1.13329

H	-2.63812	2.98747	2.76376

H	-0.9235	5.09708 -0.57392

H	-2.45313	5.04876	1.38644

C	1.86061	1.02841 -0.23198

C	2.50203	0.33522 -1.28568

C	2.57619	2.05176	0.4321

C	3.81008	0.63554 -1.63839

H	1.95108 -0.41675 -1.83772

C	3.89418	2.32736	0.09329

H	2.0939	2.59181	1.23874

C	4.51503	1.6237	-0.94412

H	4.28117	0.10567 -2.46029

H	4.4413	3.09206	0.63589

H	5.54138	1.85106 -1.21598

C	0.82281 -1.73308	0.15954

C	0.47824 -2.9633	-0.45332

C	1.99222 -1.69427	0.96722

C	1.29233 -4.07343 -0.31381

H	-0.42278 -3.01487 -1.05081

C	2.78686 -2.81934	1.12595

H	2.23328 -0.78775	1.50711

C	2.45144 -4.00856	0.47452

H	1.02709 -5.00075 -0.81148

H	3.66183 -2.77514	1.76669

H	3.07781 -4.88791	0.59066

C	-1.48867 -0.78426 -0.25232

C	-2.36856 -1.07086	0.87574

C	-1.9109	-0.88403 -1.54588

C	-3.72479 -1.40708	0.536

C	-3.29219 -1.23645 -1.88943

H	-1.24336 -0.69952 -2.38449

C	-4.16384 -1.48448 -0.76148

H	-4.39719 -1.57938	1.3718

H	-5.19494 -1.7351	-0.99363

O	-1.91026 -1.05338	2.0554

O	-3.66209 -1.2845	-3.08555
