# Supplementary material for: Intrinsic hydroquinone-functionalized aggregation-induced emission core shows redox and pH sensitivity
Source: Commun Chem. 2021 Apr 23;4:55. doi: 10.1038/s42004-021-00492-4 (PMC9814920; doi:10.1038/s42004-021-00492-4)
Supplement: Supplementary file 4 — Supplementary Data 1 [file 42004_2021_492_MOESM4_ESM.zip › Supplementary Data 3.doc]

**Cartesian Coordinates**

Optimized ground state geometry

SCF energy = -1153.184716 a.u.

C	-0.07347 -0.54209 -0.05979

C	-0.45607	0.76457 -0.06755

C	0.52543	1.86427 -0.27678

C	1.45301	1.81497 -1.33117

C	0.52019	2.9955	0.55599

C	2.36465	2.85244 -1.53001

H	1.45475	0.95501 -1.99318

C	1.43726	4.03095	0.36501

H	-0.20078	3.05488	1.36685

C	2.36536	3.96326 -0.67912

H	3.07418	2.79493 -2.35157

H	1.42737	4.88981	1.03125

H	3.07793	4.76953 -0.83164

C	-1.86354	1.19702	0.15703

C	-2.60404	0.71249	1.24803

C	-2.46937	2.13311 -0.69766

C	-3.91851	1.12887	1.46316

H	-2.14081	0.0028	1.92671

C	-3.78745	2.5443	-0.49141

H	-1.90258	2.53103 -1.53513

C	-4.51946	2.04173	0.58977

H	-4.47291	0.74322	2.31493

H	-4.24242	3.25853 -1.17296

H	-5.5439	2.36466	0.75486

C	-1.04338 -1.67012 -0.06579

C	-0.83336 -2.78237	0.76645

C	-2.15956 -1.67896 -0.92019

C	-1.7306	-3.8526	0.77405

H	0.03725 -2.79722	1.41586

C	-3.0511	-2.75126 -0.92328

H	-2.32101 -0.84016 -1.58979

C	-2.8459	-3.84151 -0.06957

H	-1.55587 -4.69691	1.43618

H	-3.90463 -2.73959 -1.59655

H	-3.54184 -4.67633 -0.0699

C	1.36322 -0.93467	0.02442

C	1.97726 -1.66244 -1.00461

C	2.09851 -0.62097	1.17517

C	3.31043 -2.05187 -0.86877

C	3.46505 -1.00088	1.37001

H	1.6083	-0.05543	1.96519

C	4.03672 -1.73883	0.28301

H	3.79224 -2.60323 -1.6771

H	5.07464 -2.05639	0.36692

O	1.23517 -1.92227 -2.1587

H	1.77811 -2.44893 -2.76543

O	4.1298	-0.70063	2.44463

Optimized S_1_ excited state geometry

SCF energy = -1153.133873 a.u.

C	-0.11075 -0.57215 -0.08412

C	-0.4966	0.79743 -0.1281

C	0.47117	1.82347 -0.51518

C	1.46833	1.57129 -1.4922

C	0.50048	3.10207	0.10141

C	2.44197	2.51777 -1.80983

H	1.45122	0.62933 -2.02944

C	1.46862	4.04767 -0.21932

H	-0.23886	3.33406	0.86262

C	2.4581	3.76563 -1.1764

H	3.18622	2.28333 -2.5682

H	1.46427	5.01007	0.28816

H	3.21659	4.50375 -1.4229

C	-1.84242	1.22473	0.27307

C	-2.55247	0.55734	1.30144

C	-2.49819	2.31546 -0.34753

C	-3.82888	0.95822	1.68686

H	-2.07821 -0.28238	1.79986

C	-3.77642	2.71721	0.0369

H	-1.99312	2.84225 -1.15236

C	-4.45805	2.04298	1.05944

H	-4.33719	0.42578	2.48805

H	-4.24838	3.55666 -0.46934

H	-5.45592	2.35291	1.3582

C	-1.01207 -1.6955	-0.24318

C	-0.70617 -2.99161	0.2729

C	-2.25018 -1.587	-0.9505

C	-1.56836 -4.07227	0.11202

H	0.22033 -3.1363	0.82159

C	-3.10401 -2.67074 -1.10532

H	-2.51928 -0.63173 -1.3884

C	-2.78384 -3.93296 -0.57504

H	-1.29024 -5.03711	0.53197

H	-4.0318	-2.53699 -1.65878

H	-3.45516 -4.77782 -0.70046

C	1.31953 -0.89525	0.23618

C	2.13059 -1.60294 -0.69588

C	1.89622 -0.54078	1.43336

C	3.48781 -1.9	-0.43586

C	3.28197 -0.82451	1.75863

H	1.312	0.00029	2.17166

C	4.05804 -1.52512	0.75655

H	4.05642 -2.42758 -1.19613

H	5.09757 -1.74482	0.97883

O	1.61848 -2.01472 -1.87578

H	0.66809 -1.77143 -1.88642

O	3.78681 -0.46576	2.86478
