# Supplementary material for: Intrinsic hydroquinone-functionalized aggregation-induced emission core shows redox and pH sensitivity
Source: Commun Chem. 2021 Apr 23;4:55. doi: 10.1038/s42004-021-00492-4 (PMC9814920; doi:10.1038/s42004-021-00492-4)
Supplement: Supplementary file 4 — Supplementary Data 1 [file 42004_2021_492_MOESM4_ESM.zip › Supplementary Data 4.doc]

**Cartesian Coordinates**

Optimized ground state geometry

SCF energy = -1152.662295 a.u.

C	-0.03617 -0.54592 -0.09398

C	-0.43454	0.76543 -0.06672

C	0.53141	1.87601 -0.28233

C	1.48982	1.81191 -1.31098

C	0.48774	3.03859	0.50644

C	2.38151	2.86116 -1.52827

H	1.53257	0.92175 -1.93051

C	1.38389	4.09012	0.2955

H	-0.25032	3.11258	1.30075

C	2.33733	4.00713 -0.7232

H	3.11292	2.78711 -2.32956

H	1.33785	4.97249	0.92955

H	3.03532	4.82345 -0.89096

C	-1.83855	1.18089	0.1967

C	-2.56488	0.65115	1.27876

C	-2.47228	2.14221 -0.61227

C	-3.88165	1.04232	1.52556

H	-2.08574 -0.07785	1.9252

C	-3.79051	2.53285 -0.3725

H	-1.92357	2.57542 -1.44444

C	-4.5056	1.98209	0.69755

H	-4.42013	0.61656	2.36893

H	-4.26137	3.26724 -1.0218

H	-5.53143	2.28709	0.88772

C	-1.02813 -1.65614 -0.13951

C	-0.86229 -2.77508	0.69341

C	-2.13877 -1.63092 -1.00055

C	-1.78579 -3.8233	0.68436

H	0.00224 -2.81229	1.34995

C	-3.0579	-2.67918 -1.02101

H	-2.27014 -0.78353 -1.66641

C	-2.89062 -3.78082 -0.17225

H	-1.64065 -4.67537	1.34452

H	-3.90328 -2.64199 -1.7043

H	-3.60745 -4.59816 -0.18596

C	1.38355 -0.94953	0.0062

C	1.95185 -1.79388 -1.00599

C	2.14266 -0.50576	1.11357

C	3.30348 -2.18331 -0.75749

C	3.49161 -0.8764	1.34841

H	1.65875	0.1469	1.84124

C	4.03397 -1.74762	0.35054

H	3.78042 -2.83226 -1.49474

H	5.06737 -2.07813	0.47506

O	1.30613 -2.1345	-2.09645

O	4.18781 -0.47534	2.39716

Optimized S_1_ excited state geometry

SCF energy = -1152.638108 a.u.

C	-0.12073 -0.53244 -0.10846

C	-0.49733	0.83604 -0.1684

C	0.46538	1.87256 -0.53332

C	1.51064	1.62317 -1.46255

C	0.45048	3.16532	0.06313

C	2.4828	2.58348 -1.74752

H	1.52837	0.67467 -1.98454

C	1.41664	4.12018 -0.22675

H	-0.32515	3.40084	0.78641

C	2.45594	3.84098 -1.13507

H	3.26497	2.34772 -2.46757

H	1.37369	5.08986	0.26635

H	3.2156	4.58658 -1.35605

C	-1.83905	1.27565	0.25226

C	-2.51996	0.65795	1.33012

C	-2.51504	2.3396	-0.3928

C	-3.78339	1.07811	1.73767

H	-2.03158 -0.16184	1.84785

C	-3.78086	2.76198	0.01195

H	-2.03369	2.82983 -1.23486

C	-4.43265	2.13603	1.08388

H	-4.26643	0.58177	2.57731

H	-4.26702	3.57929 -0.51751

H	-5.42147	2.46002	1.39848

C	-1.04459 -1.63856 -0.19746

C	-0.72084 -2.94914	0.27623

C	-2.3223	-1.52589 -0.83803

C	-1.60542 -4.01735	0.17503

H	0.24569 -3.11262	0.74208

C	-3.19664 -2.59989 -0.94331

H	-2.60731 -0.57373 -1.27175

C	-2.86673 -3.86653 -0.42863

H	-1.30621 -4.98794	0.56842

H	-4.15155 -2.45328 -1.44682

H	-3.55464 -4.70379 -0.51182

C	1.31958 -0.90629	0.13684

C	2.18045 -1.42619 -0.91878

C	1.78259 -0.88055	1.4312

C	3.53527 -1.75624 -0.53775

C	3.12522 -1.25647	1.82749

H	1.12733 -0.51575	2.2206

C	3.99026 -1.68946	0.75949

H	4.19307 -2.09445 -1.33685

H	5.01361 -1.96222	1.01121

O	1.77139 -1.63041 -2.11484

O	3.52006 -1.16543	3.04235
