# Supplementary material for: Intrinsic hydroquinone-functionalized aggregation-induced emission core shows redox and pH sensitivity
Source: Commun Chem. 2021 Apr 23;4:55. doi: 10.1038/s42004-021-00492-4 (PMC9814920; doi:10.1038/s42004-021-00492-4)
Supplement: Supplementary file 4 — Supplementary Data 1 [file 42004_2021_492_MOESM4_ESM.zip › Supplementary Data 5.doc]

**Cartesian Coordinates**

Optimized ground state geometry

SCF energy = -1152.579781 a.u.

C	-0.01795 -0.52146	0.00986
C	0.4965	0.73926 -0.00863
C	-0.38036	1.92213	0.20137
C	-1.30977	1.93123	1.25542
C	-0.26542	3.06578 -0.60268
C	-2.11477	3.04576	1.4767
H	-1.38611	1.05502	1.89214
C	-1.07419	4.17964 -0.3814
H	0.45971	3.07207 -1.41172
C	-2.00422	4.17335	0.65902
H	-2.83228	3.03327	2.29336
H	-0.98065	5.05121 -1.02527
H	-2.63661	5.04076	0.83298
C	1.94032	1.02987 -0.20945
C	2.68442	0.37929 -1.2086
C	2.59408	1.99735	0.57294
C	4.03432	0.66277 -1.39812
H	2.18973 -0.35835 -1.83123
C	3.94577	2.28003	0.3867
H	2.02798	2.52237	1.33636
C	4.67522	1.61222 -0.59889
H	4.5873	0.14371 -2.17733
H	4.43111	3.02453	1.01365
H	5.72915	1.83419 -0.74759
C	0.83301 -1.7367	0.10042
C	0.56357 -2.84558 -0.71556
C	1.89068 -1.82439	1.01877
C	1.35646 -3.99044 -0.64793
H	-0.27663 -2.7953	-1.40108
C	2.67706 -2.96985	1.09324
H	2.08018 -0.98608	1.67996
C	2.41965 -4.05688	0.25364
H	1.13768 -4.83556 -1.29652
H	3.48806 -3.01989	1.8161
H	3.03411 -4.9523	0.31256
C	-1.4764	-0.78831 -0.08839
C	-2.13101 -1.3695	1.07728
C	-2.15998 -0.54767 -1.25985
C	-3.52202 -1.72873	0.89311
C	-3.55652 -0.89339 -1.43765
H	-1.65471 -0.09323 -2.10967
C	-4.18945 -1.50647 -0.28135
H	-4.02057 -2.17141	1.75398
H	-5.23839 -1.77616 -0.3945
O	-1.5261	-1.48973	2.18252
O	-4.17397 -0.68859 -2.52094
