# Supplementary material for: Intrinsic hydroquinone-functionalized aggregation-induced emission core shows redox and pH sensitivity
Source: Commun Chem. 2021 Apr 23;4:55. doi: 10.1038/s42004-021-00492-4 (PMC9814920; doi:10.1038/s42004-021-00492-4)
Supplement: Supplementary file 4 — Supplementary Data 1 [file 42004_2021_492_MOESM4_ESM.zip › Supplementary Data 6.doc]

**Cartesian Coordinates**

Optimized ground state geometry

SCF energy = -1153.755994 a.u.

C	-0.08562 -0.59665 -0.1147
C	-0.4748	0.78299 -0.07618
C	0.47174	1.81418 -0.50387
C	1.43186	1.55214 -1.51429
C	0.52156	3.10215	0.08655
C	2.3693	2.50174 -1.89844
H	1.41588	0.57903 -1.99436
C	1.45663	4.05416 -0.30533
H	-0.18288	3.33962	0.87827
C	2.39626	3.76813 -1.30173
H	3.08527	2.25625 -2.68121
H	1.46193	5.0281	0.1824
H	3.13073	4.5109	-1.60421
C	-1.7917	1.19036	0.40975
C	-2.48317	0.43606	1.39271
C	-2.46437	2.33826 -0.0823
C	-3.74272	0.80344	1.84705
H	-2.00225 -0.45017	1.79255
 C	-3.7224	2.70797	0.38012
 H	-1.9832	2.93231 -0.85341
 C	-4.38182	1.94623	1.35128
 H	-4.2322	0.19463	2.60559
 H	-4.20159	3.59498 -0.03209
 H	-5.36811	2.23252	1.70897
 C	-1.04688 -1.66112 -0.37744
 C	-0.8926	-2.96339	0.15925
 C	-2.20528 -1.44058 -1.16359
 C	-1.82326 -3.96849 -0.07465
 H	-0.0273	-3.16864	0.78289
 C	-3.13777 -2.44428 -1.38993
 H	-2.35346 -0.45842 -1.5994
 C	-2.96201 -3.72404 -0.85002
 H	-1.66549 -4.95364	0.36275
 H	-4.01172 -2.22946 -2.00298
 H	-3.69409 -4.50871 -1.02746
 C	1.30002 -0.95123	0.21021
 C	2.05517 -1.93368 -0.4845
 C	1.98794 -0.26087	1.23224
 C	3.37225 -2.21433 -0.13213
 C	3.31464 -0.51759	1.55773
 H	1.43823	0.50663	1.77195
 C	4.02374 -1.5151	0.88971
 H	3.91871 -2.9733	-0.69378
 H	5.05739 -1.71865	1.14942
 O	1.46522 -2.56183 -1.56236
 H	2.1036	-3.20215 -1.91079
 O	3.96428	0.1793	2.5621
 H	3.38038	0.9097	2.81815
