# Supplementary material for: Intrinsic hydroquinone-functionalized aggregation-induced emission core shows redox and pH sensitivity
Source: Commun Chem. 2021 Apr 23;4:55. doi: 10.1038/s42004-021-00492-4 (PMC9814920; doi:10.1038/s42004-021-00492-4)
Supplement: Supplementary file 4 — Supplementary Data 1 [file 42004_2021_492_MOESM4_ESM.zip › Supplementary Data 7.doc]

**Cartesian Coordinates**

Optimized ground state geometry

SCF energy = -1153.475018 a.u.

C	-0.07145 -0.56442 -0.05016
C	-0.4639	0.78912 -0.0303
C	0.49697	1.84943 -0.34444
C	1.45174	1.68241 -1.37231
C	0.47158	3.06948	0.36675
C	2.35557	2.69368 -1.66533
H	1.45045	0.76773 -1.95456
C	1.39432	4.06923	0.08463
H	-0.25434	3.20637	1.16121
C	2.33686	3.88536 -0.9319
H	3.07193	2.5619	-2.46995
H	1.38156	4.99274	0.65472
H	3.05069	4.67154 -1.15759
C	-1.83333	1.1868	0.29281
C	-2.57265	0.51588	1.295
C	-2.43366	2.27851 -0.37564
C	-3.8614	0.9206	1.60978
H	-2.11499 -0.30424	1.83588
C	-3.7338	2.66197 -0.07386
H	-1.88107	2.79297 -1.1542
C	-4.44947	1.98771	0.92103
H	-4.41134	0.4101	2.39381
H	-4.19333	3.48442 -0.61256
H	-5.4625	2.29493	1.16181
C	-1.03992 -1.64909 -0.19905
C	-0.85759 -2.85524	0.51279
C	-2.15033 -1.53153 -1.06529
C	-1.7684	-3.89432	0.38375
H	-0.01426 -2.94888	1.18943
C	-3.04588 -2.58111 -1.2064
H	-2.28223 -0.62393 -1.64289
C	-2.86298 -3.76212 -0.47818
H	-1.63342 -4.80701	0.95544
H	-3.88568 -2.48666 -1.8873
H	-3.57128 -4.57819 -0.58289
C	1.3338	-0.93074	0.12337
C	1.95741 -1.89341 -0.72133
C	2.10295 -0.33944	1.14748
C	3.30011 -2.23875 -0.51471
C	3.42712 -0.69568	1.35014
H	1.63011	0.39189	1.79545
C	4.02645 -1.6583	0.50903
H	3.77318 -2.96719 -1.16819
H	5.0634	-1.92587	0.6808
O	1.24322 -2.38084 -1.76176
H	1.78927 -2.9936	-2.28105
O	4.20948 -0.18391	2.33114
H	3.71704	0.47517	2.84609
