# Supplementary material for: Intrinsic hydroquinone-functionalized aggregation-induced emission core shows redox and pH sensitivity
Source: Commun Chem. 2021 Apr 23;4:55. doi: 10.1038/s42004-021-00492-4 (PMC9814920; doi:10.1038/s42004-021-00492-4)
Supplement: Supplementary file 4 — Supplementary Data 1 [file 42004_2021_492_MOESM4_ESM.zip › Supplementary Data 8.doc]

**Cartesian Coordinates**

Optimized ground state geometry

SCF energy = -1153.180018 a.u.

C	-0.70645 -0.40501	0.0617
C	-0.02996	0.7505	-0.13179
C	-0.73613	2.06755 -0.24143
 C	-1.20646	2.6907	0.92433
 C	-0.93187	2.7138	-1.47092
 C	-1.86374	3.922	0.86436
 H	-1.0623	2.1944	1.88031
 C	-1.58619	3.9467	-1.5353
 H	-0.59626	2.2362	-2.38737
 C	-2.05528	4.55593 -0.3673
 H	-2.22775	4.38405	1.77851
 H	-1.73724	4.42632 -2.49908
 H	-2.56776	5.51298 -0.41708
 C	1.46225	0.81334 -0.12496
 C	2.17434	1.47132 -1.14138
 C	2.18634	0.2434	0.93718
 C	3.57047	1.53042 -1.11807
 H	1.63828	1.93243 -1.96483
 C	3.58112	0.30806	0.96382
 H	1.64251 -0.2438	1.74128
 C	4.28131	0.94669 -0.0652
 H	4.10075	2.03333 -1.92298
 H	4.12215 -0.13976	1.79399
 H	5.36704	0.99457 -0.04366
 C	-2.19845 -0.44955	0.11047
 C	-2.84339 -1.02485	1.22013
 C	-2.98545	0.02391 -0.95151
 C	-4.2371	-1.08947	1.27666
 H	-2.22709 -1.39759	2.03395
 C	-4.38014 -0.04975 -0.89862
 H	-2.50195	0.44725 -1.82563
 C	-5.01331 -0.60089	0.21926
 H	-4.71969 -1.52459	2.14872
 H	-4.97031	0.32147 -1.73302
 H	-6.09813 -0.65473	0.26366
 C	-0.02657 -1.7349	0.17591
 C	0.24375 -2.31297	1.46712
 C	0.30777 -2.40275 -1.0079
 C	0.86557 -3.599	1.43351
 C	0.9204	-3.655	-0.98554
 H	0.09368 -1.93455 -1.96685
 C	1.19619 -4.25474	0.24484
 H	1.0969	-4.06959	2.38751
 H	1.67395 -5.23414	0.28008
 O	-0.02876 -1.69795	2.57895
 O	1.2301	-4.25513 -2.20871
 H	1.65627 -5.10974 -2.03937
